# The effectiveness of tissue-perfusion-guided resuscitation in shock: A systematic review and meta-analysis

**DOI:** 10.1016/j.aicoj.2026.100106

**Published:** 2026-06-25

**Authors:** Tamás Tóth, Patricia Schneidereit, Julia Hollosi, Dávid Lackó, Bence Szabó, Daniel Louis Albert, Caner Turan, László Zubek, Péter Hegyi, Zsolt Molnar

**Affiliations:** a Centre for Translational Medicine, Semmelweis University, Budapest, Hungary; b Department of Anaesthesiology and Intensive Therapy, Bajcsy-Zsilinszky Hospital, Budapest, Hungary; c Department of Interventional Radiology, Heart and Vascular Centre, Semmelweis University, Budapest, Hungary; d Department of Anesthesiology and Intensive Therapy, Semmelweis University, Budapest, Hungary; e Institute for Translational Medicine, Medical School, University of Pécs, Pécs, Hungary; f Institute of Pancreatic Diseases, Semmelweis University, Budapest, Hungary; g Division of Gastroenterology, First Department of Medicine, Medical School, University of Pécs, Pécs, Hungary; h Department of Anesthesiology and Intensive Therapy, Poznan University of Medical Sciences, Poznan, Poland

**Keywords:** Shock, Tissue perfusion, Capillary refill time, Fluid resuscitation, Meta-analysis

## Abstract

Resuscitation from shock generally targets macrohemodynamic parameters. However, this approach may not be followed by improved microcirculation and carries a risk of fluid overload. We aimed to evaluate the efficacy of tissue-perfusion-guided therapy (TP-GT) compared with standard care in adult patients with shock. Following the PRISMA 2020 guidelines, we searched MEDLINE, Embase, and CENTRAL on October 26, 2025, for randomized controlled trials (RCTs) comparing TP-GT to standard therapy. The protocol was prospectively registered on PROSPERO (CRD420251163043). The primary outcomes were 30-day and 90-day mortality. Secondary outcomes included intensive care unit length of stay (ICU LOS), hospital length of stay (HLOS), fluid administration volumes, fluid balances and organ support requirements. The meta-analyses were performed using the random-effect model, and the risk of bias was assessed using the RoB2 tool. Eight RCTs were analyzed, comprising 2,394 patients. TP-GT did not significantly reduce either 30-day mortality (Risk Ratio [RR] 0.96; 95% CI 0.83–1.10; *P* = 0.49) or 90-day mortality (RR 0.94; 95% CI 0.82–1.07; *P* = 0.17) compared to standard care. However, patients managed with TP-GT were associated with a shorter ICU LOS (Mean Difference [MD] −0.72 day; 95% CI −1.42 to −0.01; P = 0.048), although this finding proved fragile in sensitivity analyses. Furthermore, TP-GT significantly reduced the volume of fluid administered through the first 6–8 h of resuscitation (MD −466.95 mL; 95% CI −917.17 to −16.72; P = 0.046). It promotes personalized fluid stewardship by significantly reducing early fluid administration and may be associated with shorter ICU LOS.

## Highlights

• Our findings showed no difference in mortality between the tissue-perfusion-guided therapy (TP-GT) and non-TP-GT groups.
• TP-GT is associated with a shorter, albeit fragile, ICU LOS.
• TP-GT led to a significant reduction in the volume of fluid administered during the first 6–8 h of resuscitation.

## Introduction

Shock is a life-threatening, generalized form of acute circulatory failure characterized by inadequate oxygen delivery to the cells [1]. It remains a challenge in critical care medicine, affecting approximately 10.4% of the intensive care population and associated with a mortality rate of approximately 38% [2]. In the acute phase, hemodynamic instability is the main cause of death [3].

Current international guidelines strongly recommend immediate resuscitation to achieve macrohemodynamic goals [4]. However, correcting macrocirculation may not be followed by the restoration of microcirculation [5]. The current large-scale, multicenter ANDROMEDA SHOCK-2 trial [6] has renewed interest in tissue-perfusion-guided strategies by demonstrating benefits in composite outcomes for CRT-guided therapy compared to standard care. However, current resuscitation therapies and treatment protocols in clinical trials seldom include assessment of microcirculation or peripheral perfusion indices as therapeutic targets. It is also important to note that fluid overload is a common feature in both clinical practice and studies and is strongly associated with increased mortality [7]. The so-called “Goal-directed therapies” almost explicitly include macrohemodynamic variables as therapeutic goals, which may inadvertently worsen fluid balance, highlighting the need for more precise hemodynamic management strategies to improve outcomes [8,9].

To evaluate tissue perfusion at the bedside, clinicians can use a wide range of tools that incorporate multiple diagnostic modalities and mechanistic proximity to the microcirculation. These include bedside clinical surrogates of peripheral perfusion, such as capillary refill time (CRT) and skin mottling, as well as direct instrumental assessments of microvascular flow, such as intravital video microscopy, near-infrared spectroscopy, and laser Doppler flowmetry [10]. While clinical markers such as CRT have prognostic relevance, they are not physiologically equivalent to direct visualization of the microcirculation. Nevertheless, all of these approaches share the same physiological rationale of helping to restore tissue perfusion during hemodynamic management. Based on this, we aimed to assess the general impact of these therapeutic approaches in patients treated for shock compared to those receiving standard care.

## Methods

This work was carried out as part of the Systems Education Program [11] at Semmelweis University and conducted within the Translational Medicine (TM) Cycle Framework by the Academia Europaea [12].

We reported our systematic review and meta-analysis in accordance with the PRISMA 2020 guidelines [13], following the Cochrane Handbook [14]. The study protocol was registered with PROSPERO (registration number CRD420251163043). This review was conducted in full adherence to the registered protocol, and no amendments were made after initial registration.

### Eligibility criteria

We included randomized controlled trials (RCTs) and secondary analyses of RCTs that focused on (P) adult patients (>18 years) experiencing any form of shock, including septic, cardiogenic, hypovolemic or mixed types. The intervention was defined as (I) tissue-perfusion-guided therapy (TP-GT), where resuscitation was directed by assessment of tissue perfusion. Eligible assessment methods encompassed CRT, perfusion index (PI), video microscopy techniques (such as Sidestream Dark-Field microscopy), mottling score, laser Doppler flowmetry, and near-infrared spectroscopy. The (C) comparator was standard shock therapy guided by conventional parameters. The primary outcome (O) was mortality at 30 and 90 days, as well as ICU mortality. Eligible secondary outcomes included the Sequential Organ Failure Assessment (SOFA), Acute Physiology and Chronic Health Evaluation (APACHE) score, lactate change, urine output, renal replacement therapy, mechanical ventilation, vasopressor and any other extracorporeal support requirements, occurrence of any adverse events, fluid balance and fluid administration volumes, changes in cardiac output, central venous pressure, and central venous oxygen saturation, as well as ICU and hospital length of stay. Studies were excluded if they involved pediatric patients, animal models, or healthy volunteers.

### Information sources

Our systematic search was conducted on October 26, 2025 across the following electronic databases: MEDLINE (via PubMed), Embase, and the Cochrane Central Register of Controlled Trials (CENTRAL). To ensure completeness, we performed forward and backward citation searches by screening the reference lists of the included articles [15].

### Search strategy

In our search process, we employed a three-domain search strategy (Additional file, Table S1). The first domain concentrated on clinical conditions associated with acute circulatory failures. The second domain was dedicated to studies that directly assessed tissue perfusion, and the third domain focused on randomized controlled trials. We did not use any restrictions on the search criteria; neither language nor date limitations were applied.

### Selection process

The selection was performed by two independent authors (T.T. and P.S.) after removing duplicates. Disagreements were resolved during a conflict meeting with two other investigators (D.L. and J.H.). The inter-reviewer agreement was assessed using Cohen’s Kappa [16], with values above 0.8 deemed acceptable for both title and abstract selection (0.846) and full-text selection (0.816).

### Data collection process

From the eligible articles, data were independently collected by two authors (T.T. and P.S.) and entered into a dedicated data table. To prevent population overlap and the risk of double-counting, two post hoc analyses [17,18] were excluded from the statistical analysis and were utilized solely for the systematic review.

### Data items

The following data items were extracted: year of publication, country of study, study period, age and sex of patients, number of patients, shock types, method of TP-GT, baseline SOFA and APACHE II scores, mortality at 30 days, mortality at 90 days, ICU mortality, ICU LOS, hospital length of stay, 24 -h fluid balance, fluid administration during the first 6–8 h, the need for renal replacement therapy, mechanical ventilation-free days, vasopressor-free days and changes in SOFA score.

### Study risk of bias assessment

Two authors (T.T. and D.L.A.) performed the risk of bias assessment independently using the Cochrane RoB-2 tool for RCTs [19]. The certainty of evidence for each outcome was evaluated using the GRADE system via GRADE-Pro software [20]. Disagreements were resolved during a conflict meeting with two other investigators (D.L. and J.H.).

### Synthesis methods

This meta-analysis was conducted using both qualitative and quantitative synthesis methods. Meta-analysis was conducted only when at least three studies reported the same outcome. A random-effects model was applied to account for variability among studies. For dichotomous outcomes, risk ratios (RR) with 95% confidence intervals (CI) were reported. For continuous outcomes, mean differences (MDs) with 95% CIs were reported, depending on how the data were presented in the included studies. Means or medians for individual groups were also presented for clarity. When studies reported medians and quartiles, we used the methodology implemented in the meta package [21,22] to estimate the mean and standard deviations. When the exact outcomes were not reported in the articles or supplementary materials but could be calculated, we calculated them. Heterogeneity among studies was assessed using Higgins and Thompson’s I² statistic and tau-square (τ²) variance measures [23], following Cochrane Collaboration guidelines [14]. Statistical significance was determined when the confidence interval did not include the null value. Findings were summarized graphically using forest plots, and prediction intervals were reported where appropriate [24]. Small study publication bias was assessed by visual inspection of Funnel plots. The inclusion of Egger’s test [25] for MD, or Harbord (modified Egger’s) test [26] for RR effect size was planned, but due to the low number of included studies, they were not performed. All statistical analyses were performed with R [27] using the meta [28] package for basic meta-analysis calculations and plots, and dmetar [29] package for additional influential analysis calculations and plots.

## Results

### Search and selection

**Fig. 1:**
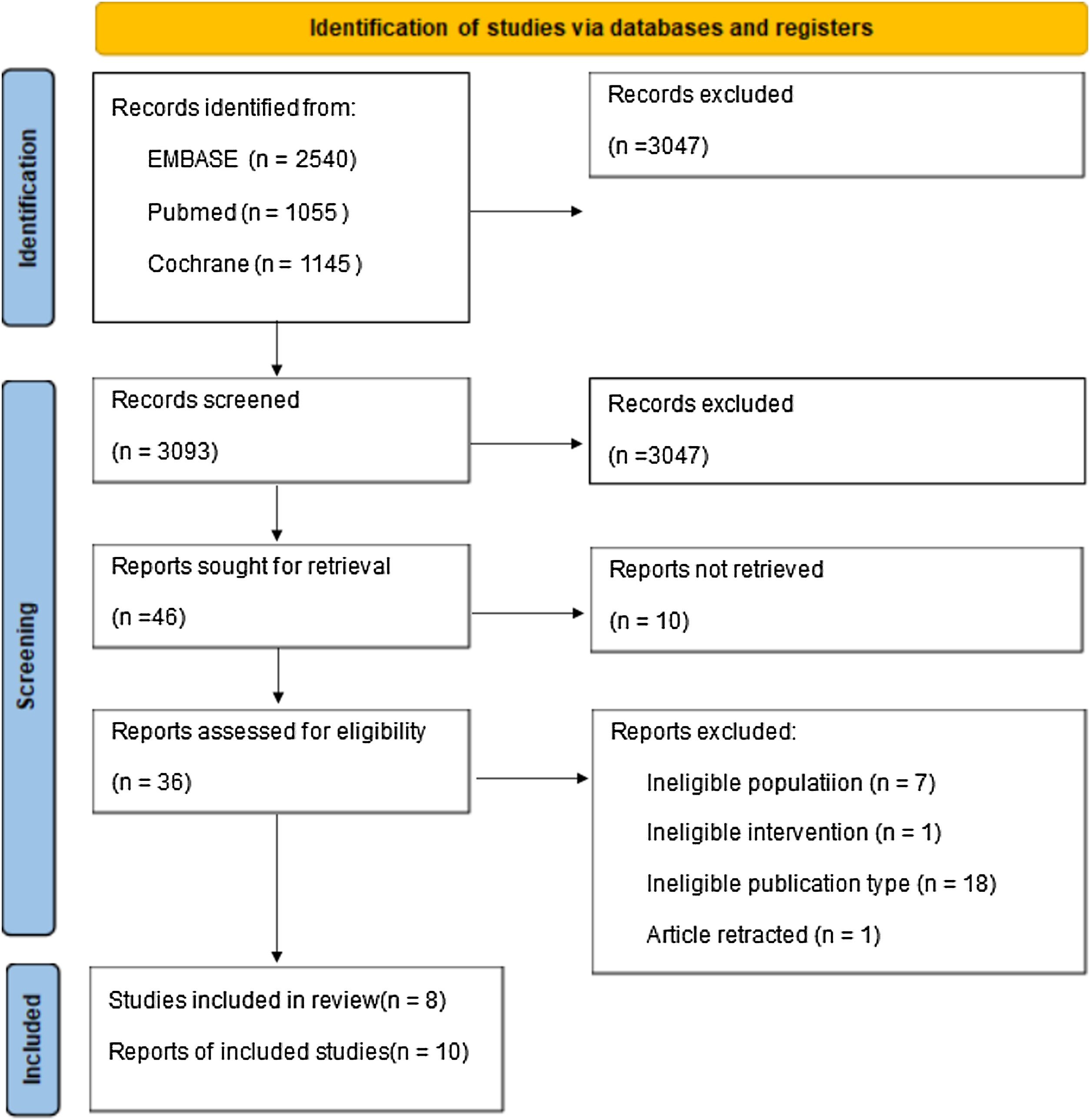
PRISMA 2020 flow diagram.

Altogether, 4,740 studies were identified by our search in MEDLINE (via PubMed), Embase, and the Cochrane Central Register of Controlled Trials (CENTRAL) databases, and a further 1,026 were screened during forward and backward citation searches. Out of the 46 articles considered for full-text selection, eight randomized controlled trials [6,[30], [31], [32], [33], [34], [35], [36]] and two post hoc analyses [17,18] were found to be eligible (Fig. 1).

### Basic characteristics of included studies

We included eight RCTs (n = 2,394) published between 2015 and 2025, primarily enrolling patients with septic shock in intensive care units [6,[30], [31], [32], [33], [34], [35], [36]]. Interventions guided resuscitation using CRT (n = 3) [6,31,32], sublingual microscopy (n = 2) [33,35], perfusion index (n = 1) [34], or composite tissue perfusion strategies (n = 2) [30,36]. Control groups received standard care focused on macrohemodynamic targets (e.g., MAP) and on lactate normalization. Baseline characteristics of the enrolled studies are described in Table 1.

**Table 1:** Baseline Characteristics of Included Randomized Controlled Trials.

Study, Year	Country (No. of Sites)	Study Design	Population (N)	Shock Type	Intervention Group (TP-GT) Protocol	Control Group Protocol	Age, years	Female Sex, No. (%)	Severity of Illness (Baseline)
Van Genderen et al., 2015	Netherlands (1)	Single-center RCT	30	Septic	PPTFM: Targeted CRT <2 s, PI > 1.4, StO2 and normal skin temp.	Standard Care	63 (58–73)*	12 (40%)	SOFA: 11 (3.9)
Hernández et al., 2019	5 Countries (28)	Multicenter RCT	424	Septic	CRT-Targeted: CRT normalization (<3 s) every 30 min.	Lactate-Targeted: Targeting lactate normalization or 20% decrease every 2 h.	62 (17)	226 (53%)	SOFA: 10 (3)
Castro et al., 2020	Chile (2)	Multicenter RCT	42	Septic	CRT-Targeted: Targeted CRT normalization (<3 s).	Lactate-Targeted: Targeted lactate normalization or 20% decrease.	58 (45–75)*	24 (57%)	SOFA: 11 (8–14)*
Zhou et al., 2021	China (1)	Single-center RCT	31	Septic	POEM-Guided: Targeted POEM score >3.0.	Standard care	58 (18)	9 (29%)	NI
Lin et al., 2022	China (1)	Single-center RCT	65	Septic	PI-Guided: Fluid resuscitation targeting Perfusion Index (PI) >1.4.	Standard Care	74 (12)	27 (42%)	APACHE II: 26.6 (6)
Bruno et al., 2023	Germany (5)	Multicenter RCT	141	Mixed**	SDF-Guided: Treatment guided by sublingual microcirculation parameters (SDF imaging).	Standard Care	69 (18)	40 (28%)	SOFA: 8 (5)
Hernández et al., 2025	19 Countries (86)	Multicenter RCT	1467	Septic	CRT-PHR: Resuscitation targeting CRT normalization (<3 s).	Usual Care	66 (52−77)*	636 (43%)	SOFA: 8 (7–11)*
Pettilä et al., 2025	3 Countries (3)	Multicenter RCT	194	Septic	TTP Strategy: Targeted Tissue Perfusion (CRT, mottling, temp, lactate) allowing lower MAP (50–65 mmHg).	Standard Care	62 (14)	112 (58%)	SOFA: 8.9 (2.5)

Abbreviations: NI, No Information; APACHE II, Acute Physiology and Chronic Health Evaluation II; CRT, Capillary Refill Time; CRT-PHR, Capillary Refill Time-Personalized Hemodynamic Resuscitation; MAP, Mean Arterial Pressure; TP-GT, Tissue-Perfusion-Guided Therapy; PI, Perfusion Index; POEM, Point of Care Microcirculation score; PPTFM, Peripheral Perfusion-Targeted Fluid Management; RCT, Randomized Controlled Trial; SDF, Sidestream Dark Field imaging; SOFA, Sequential Organ Failure Assessment; TTP, Targeted Tissue Perfusion.

Notes: Data are presented as Mean (SD) or *Median (Interquartile Range) unless otherwise indicated. ** Mixed shock population in Bruno et al. (2023) included Cardiogenic (55%), Post-cardiac surgery (19%), Septic shock (16%), Hemorrhagic shock (7%) and Neurological shock (3%). All other studies exclusively enrolled patients with Septic Shock.

### Mortality

**Fig. 2:**
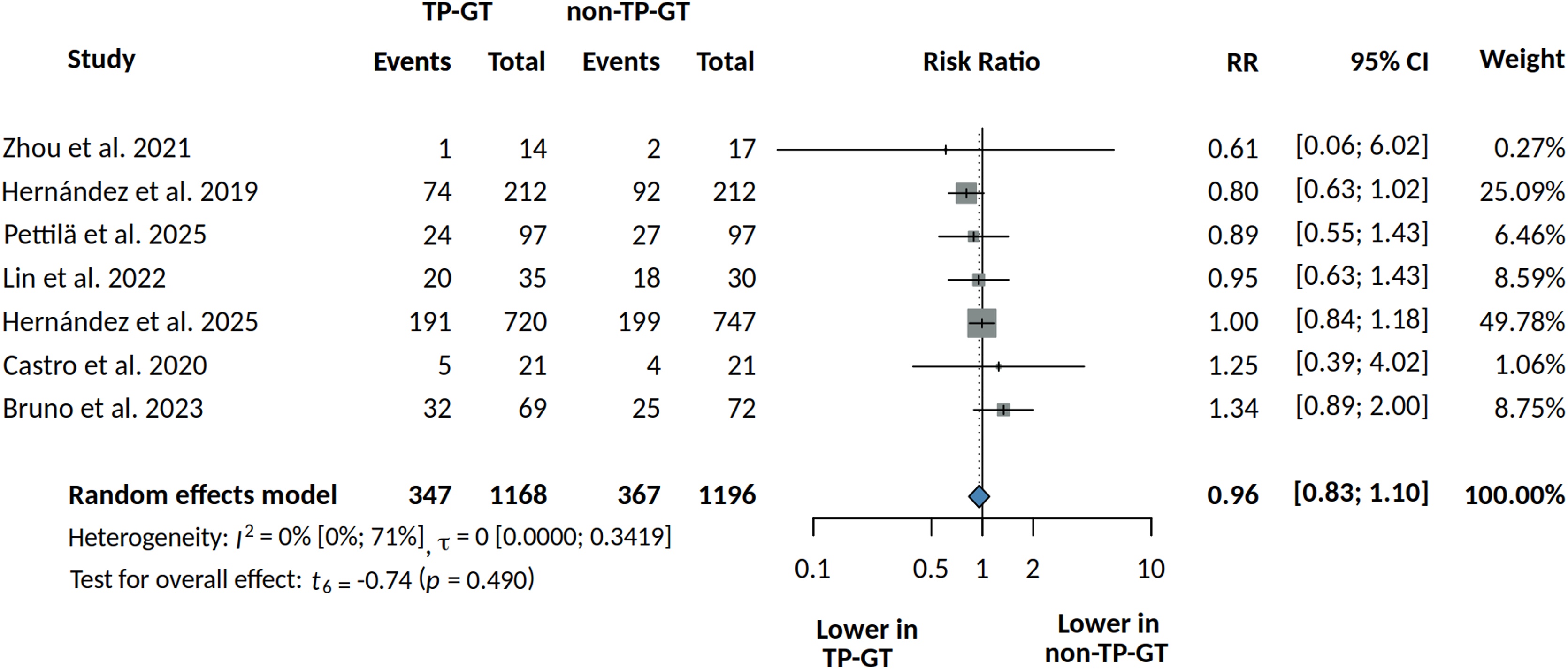
Forest plot comparing 30-day mortality between TP-GT and standard care in critically ill patients. The size of the squares is proportional to the weight of each study in the meta-analysis. The horizontal lines represent 95% CIs. The diamond represents the overall pooled effect, calculated using a Mantel–Haenszel random-effects model. Statistical heterogeneity was assessed using the I^2^ statistic test. CI = confidence interval; RR = risk ratio

Regarding the primary outcome, the pooled analysis of seven trials [6,[31], [32], [33], [34], [35], [36]] (n = 2,364) demonstrated no statistically significant reduction in 30-day mortality with TP-GT compared with standard care (RR 0.96; 95% CI 0.83–1.10; *P* =  0.49; Fig. 2). This lack of survival benefit was consistent across extended follow-up; among the subgroup of studies [6,31,36] reporting 90-day mortality (3 trials; n = 2,081; RR:0.94; 95% CI 0.82–1.07; *P* =  0.17; Additional file, Figure S2). Furthermore, sensitivity analyses excluding the heavily weighted ANDROMEDA-SHOCK-2 trial [6] or isolating a pure septic shock population by excluding the mixed-shock trial (DAMIS trial [35]) did not alter the non-significant mortality outcomes (Additional file, Figures S4 and S6).

### Length of stay

**Fig. 3:**
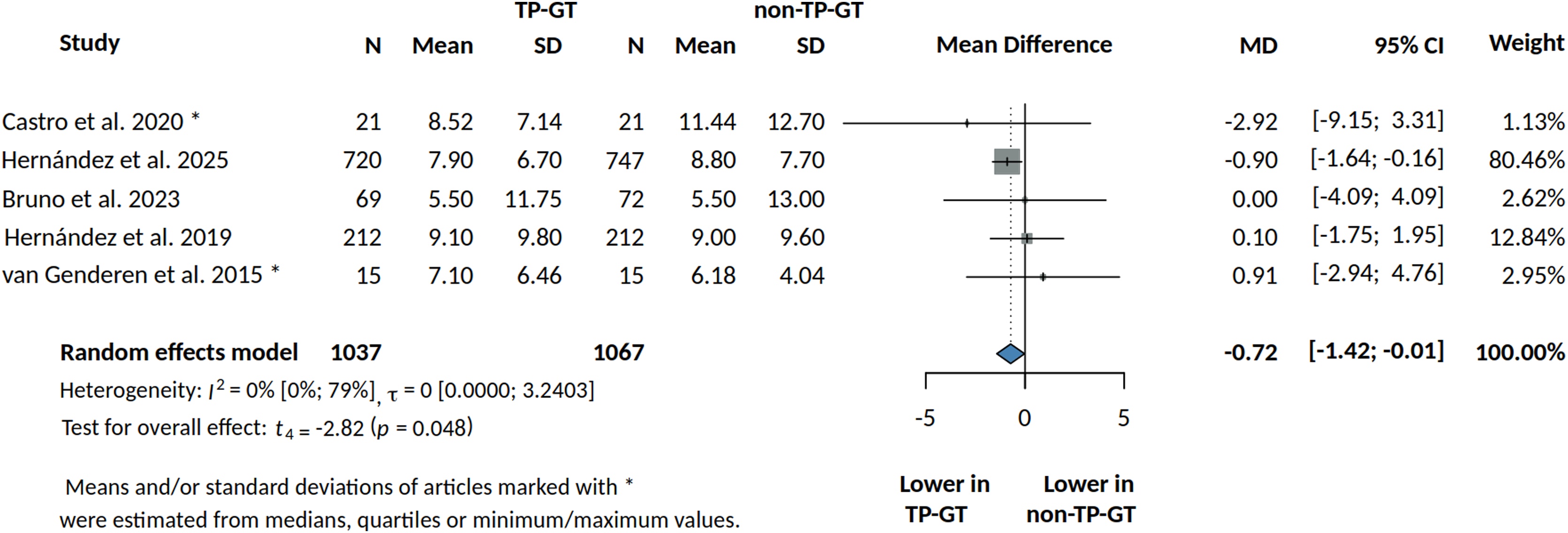
Forest plot comparing ICU LOS between TP-GT and standard care. The size of the squares is proportional to the weight of each study in the meta-analysis. The horizontal lines represent 95% CIs. The diamond represents the overall pooled effect, calculated using an inverse variance random-effects model. Means and/or standard deviations for select studies were estimated from medians, quartiles, or minimum/maximum values. Statistical heterogeneity was assessed using the I^2^ statistic test. CI = confidence interval; ICU = intensive care unit; MD = mean difference

Length of stay was reported by the same five trials [6,[30], [31], [32],^35^]. Regarding ICU LOS (n = 2,104), TP-GT was associated with a statistically significant reduction of 0.72 days compared with standard care (MD −0.72; 95% CI −1.42 to −0.01; P = 0.048; Fig. 3). However, data from the same studies indicated that this benefit did not translate into a reduction in hospital length of stay. The mean difference was 0.19 days (95% CI −5.57 to 5.96; P = 0.93; Additional file, Figure S11). However, sensitivity analyses revealed that this reduction in ICU LOS is highly fragile. This outcome lost statistical significance when either the heavily weighted ANDROMEDA-SHOCK-2 [6] trial or the mixed-shock trial (DAMIS trial [35]) was excluded from the pooled analysis (Additional file, Figures S13 and S15).

### Early fluid administration

**Fig. 4:**
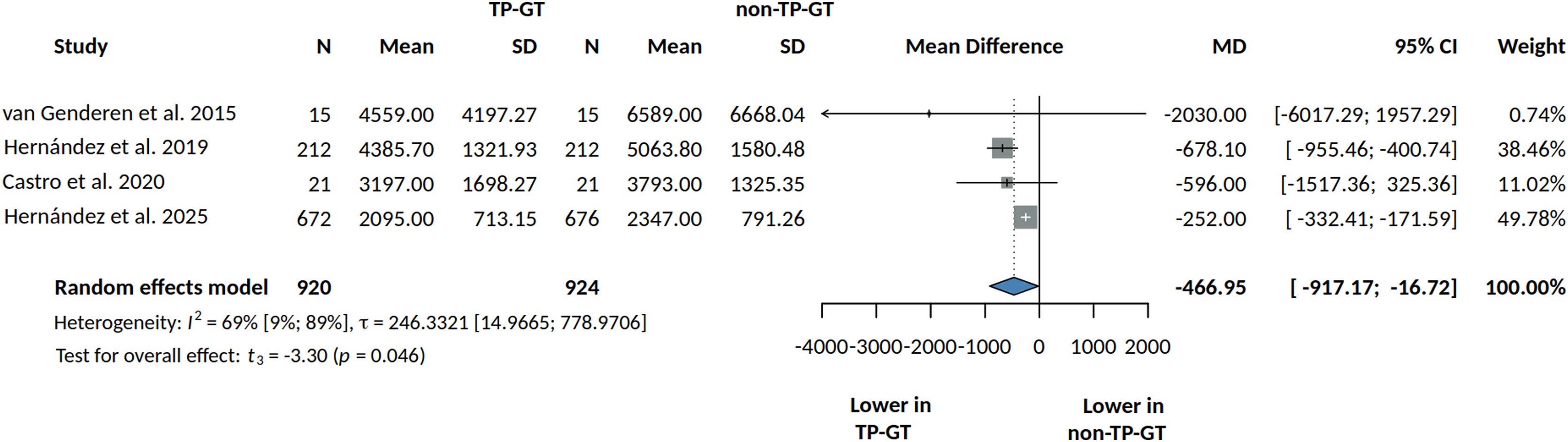
Forest plot comparing total fluid administration during the first 6 to 8 h of resuscitation between TP-GT and standard care. The size of the squares is proportional to the weight of each study in the meta-analysis. The horizontal lines represent 95% CIs. The diamond represents the overall pooled effect, calculated using an inverse variance random-effects model. Statistical heterogeneity was assessed using the I^2^ statistic test. CI = confidence interval; MD = mean difference.

Early fluid administration volumes (up to 6–8 h) were analyzed in four trials (n = 1,844), with additional pre-randomization volumes and missing data obtained via author correspondence where necessary. The TP-GT group received significantly less fluid during this early resuscitation phase compared with the standard care group (MD −466.95 mL; 95% CI −917.17 to −16.72; P = 0.046; Fig. 4). However, for 24-h fluid balance, no statistically significant difference was observed between the intervention and control groups (MD −211.15 mL; 95% CI −463.89 to 41.60; P = 0.08; Additional file, Figure S20). This reduction in early fluid administration remained statistically significant even after excluding the heavily weighted ANDROMEDA-SHOCK-2 trial [6] (Additional file, Figure S22). However, when restricting the subgroup analysis exclusively to CRT-guided interventions (ANDROMEDA-1 [31], ANDROMEDA-2 [6], and Castro et al. [32]), the difference in early fluid intake lost statistical significance (Additional file, Figure S24).

### Organ support

Regarding organ support, the point estimates consistently favored the TP-GT group, although these differences did not reach statistical significance. Specifically, no significant benefit was observed for vasopressor-free days (MD 1.28; 95% CI −2.01–4.58; P = 0.24; Additional file, Figure S26), mechanical ventilation-free days (MD 0.82; 95% CI −0.84 to 2.48; P = 0.21; Additional file, Figure S28), or the need for renal replacement therapy (RR 0.85; 95% CI 0.64–1.12; P = 0.15; Additional file, Figure S30). Consistent with these findings, the reduction in SOFA score at 72 h was numerically greater in the intervention group, but this result was not statistically significant (MD=-0.65; 95% CI = −1.8 to 0.5; P = 0.14; Additional file, Figure S32).

### Risk of bias assessment

The risk of bias in included studies ranged from low to some concern, with no study assessed as high risk. For randomization, most studies showed low risk of bias, including those with some concern due to simple randomization. Evaluation of intervention deviations raised concerns in several studies, mainly because the studies were open-label, as the nature of the intervention precluded blinding. Bias due to missing outcomes was low in all studies, with either no missing data or acceptably low amounts reported. Bias in measuring objective endpoints such as mortality was predominantly low risk. However, subjective outcomes such as length of stay and organ support needs, which depend on clinicians' decisions, were often judged as "some concern" due to the unblinded design. Detailed domain-level judgments for each study across outcomes are provided in the supplementary material (Additional file, Figure S34-S43).

The certainty of evidence ranged from low to moderate. Evidence for 90-day mortality, ICU LOS, vasopressor-free days, and early (6–8 h) fluid administration was rated as moderate certainty, downgraded one level due to either serious imprecision or serious inconsistency. All other outcomes, such as 30-day mortality, hospital length of stay, mechanical ventilation-free days, need for renal replacement therapy, 72-h SOFA score change, and 24-h fluid balance, were evaluated as low certainty and downgraded by two levels due to serious inconsistency and imprecision. Notably, no outcomes were downgraded for risk of bias, indirectness, or other considerations. Detailed GRADE assessment [20] can be found in the Additional file (Figure S44).

## Discussion

### Mortality

Our findings indicate no significant difference in mortality outcomes between the TP-GT group and the control group. An explanation could be that although this analysis addressed various types of shock, the vast majority of patients (95%) treated in the analyzed trials were suffering from septic shock. Septic shock is a heterogeneous syndrome rather than a single disease, implying that the diverse patient population may include phenotypes that benefit from this type of treatment as well as those for whom it may be detrimental [37]. The use of mortality as a primary outcome measure in critical care settings, particularly in cases of septic shock, has been a subject of extensive debate [38,39]. The evaluation of survival in these cases is highly affected by disease heterogeneity and countless clinical decisions, thus it is highly unlikely that changing just one aspect of treatment could cause a measurable reduction in mortality [40,41].

### Clinical recovery and fluid stewardship

Our primary pooled analysis suggested a significantly shorter ICU LOS for the intervention group. However, our sensitivity analyses (Additional file, Figures S13 and S15) revealed this finding to be highly fragile, losing statistical significance when a specific landmark [6] or mixed-shock trial [35] was excluded. This elucidates two critical points: first, subjective outcomes such as ICU discharge are highly vulnerable to co-intervention and discharge-decision bias in unblinded trials; and second, our pooled estimates are heavily driven by a few studies. The lack of robust differences in organ failure resolution suggests that organ recovery depends on complex factors beyond early hemodynamic management.

Despite these limitations, the observed reduction in ICU LOS is likely physiologically linked to the significant decrease in early fluid administration associated with TP-GT. This potential effect is probably rooted in the concept of hemodynamic coherence [5]. Because traditional resuscitation targets, such as serum lactate normalization, are slow and influenced by other metabolic pathways, blindly chasing lactate clearance often leads to overresuscitation [42]. In contrast, tissue perfusion parameters like CRT can quickly indicate microcirculatory changes [43]. Rapid responsiveness is crucial during treatment, and tissue perfusion assessments can serve as potential "stop signs" to mitigate complications of unnecessary fluid overload, such as tissue edema, prolonged mechanical ventilation, and other organ dysfunctions [7,44]. Consequently, mitigating these complications potentially facilitates faster clinical stabilization and readiness for discharge.

Personalized, fluid-sparing approaches in intensive therapy are well supported by recent literature [45]. Randomized controlled trials with large patient numbers, such as the CLASSIC trial [46] and the CLOVERS trial [47], aimed to evaluate restrictive fluid therapies in septic shock and found no difference in mortality, however, increasing evidence highlights the danger of fluid overload [8,44]. While the 2021 Surviving Sepsis Campaign guidelines [4] recommend using dynamic hemodynamic parameters and lactate measurements to guide fluid administration, fluid responsiveness doesn’t equate to the necessity of fluid [48]. These findings strongly align with the shift toward restrictive fluid stewardship, or rather, personalized fluid management in intensive care settings.

Two post hoc analyses from the ANDROMEDA-SHOCK [31] trial provide crucial context for our results. A Bayesian reanalysis by Zampieri et al. [17] demonstrated a very high probability of lower mortality and faster resolution of organ dysfunction with a peripheral perfusion-oriented strategy. Kattan et al. [18] showed that targeting lactate normalization in septic shock patients who already have a normal CRT leads to excessive therapeutic interventions, greater organ dysfunction, and higher mortality. This highlights a critical pitfall: hyperlactatemia in sepsis is frequently non-hypoperfusion-related [49], and chasing lactate clearance without evaluating peripheral tissue perfusion carries a risks of over-resuscitation. In contrast, bedside tissue-perfusion targets, such as CRT, provide a rapid feedback loop to safely withhold further fluids, thereby directly explaining the fluid-sparing effect observed in our meta-analysis [32,43].

### Strengths and limitations

To the best of our knowledge, this is the first meta-analysis to investigate the effect of TP-GT in patients with shock. A major strength of this study is its high clinical relevance and timeliness, as it provides evidence for personalized hemodynamic resuscitation by directly addressing the well-known physiological gap between macrohemodynamic correction and the restoration of tissue perfusion. Furthermore, by focusing exclusively on randomized controlled trials, we ensured the highest quality of analyzed evidence. Additional strengths include our highly sensitive and comprehensive search strategy, rigorous overall methodology, and strict adherence to the pre-registered PROSPERO analysis plan. Finally, our robust approach followed current methodological standards, utilizing validated frameworks such as the PRISMA 2020 guidelines [13], the Cochrane RoB-2 tool [19] for structured risk-of-bias assessment, and the GRADE system [20] to formally evaluate the certainty of the evidence.

However, several limitations of this study must be acknowledged to contextualize our findings appropriately.

First, although all included interventions share the same physiological goal of restoring tissue perfusion, there is conceptual and methodological heterogeneity among the assessed interventions, emphasizing that TP-GT is not a monolithic approach. The included trials used a wide range of tools, from bedside clinical techniques (e.g., CRT) to direct instrumental visualization (e.g., sublingual video microscopy). While CRT provides rapid, low-cost physiological feedback, direct instrumental techniques offer precise microvascular assessments, however they are hindered by higher costs, limited bedside availability, and the need for specialized training [50,51]. Second, the included populations and control interventions varied across trials. Seven studies exclusively enrolled septic shock patients, whereas the mechanistically distinct DAMIS trial [35] introduced pathophysiological diversity with a predominantly cardiogenic shock cohort. Furthermore, control groups ranged from strict lactate-targeted protocols to standard care.

Third, our pooled estimates are heavily driven by a few highly weighted landmark studies, especially the ANDROMEDA-SHOCK-2 trial. Consequently, the observed clinical benefits, most notably the reduction in early fluid administration, predominantly reflect the efficacy of peripheral perfusion-guided protocols in septic shock, rather than the broad application of direct microcirculatory visualization across all shock states. The small number of eligible RCTs (n = 8) also severely limited our statistical power. For instance, restricting the subgroup analysis exclusively to CRT-guided interventions caused the fluid-sparing effect to lose statistical significance. Similarly, excluding either the ANDROMEDA-SHOCK-2 [6] or the DAMIS trial [35] eliminates the significant reduction in ICU LOS, underscoring that our summary effects are highly reliant on these individual trials. Finally, blinding is virtually impossible in clinical trials involving real-time hemodynamic monitoring tools. This open-label design may introduce performance bias in the management of the intervention group and discharge-decision bias. Consequently, subjective secondary endpoints, particularly ICU LOS, remain highly vulnerable to these co-interventions and must be interpreted with caution.

### Implication for practice and research

Implementing scientific findings into clinical practice is crucial [52]. While our results demonstrate that TP-GT represents a promising, physiologically relevant approach that may protect patients from fluid overload, recommending its immediate integration into critical care resuscitation guidelines is premature. The pooled evidence did not demonstrate a difference in mortality or clear signals of harm compared with standard care. Therefore, future large-scale randomized trials and multicenter registries are needed to identify the most reliable and cost-efficient monitoring tools and to determine the specific biological shock phenotypes most likely to derive a definitive survival benefit from tissue-perfusion monitoring.

## Conclusion

TP-GT showed no difference in short- or long-term mortality compared with standard care. Although it did not improve survival, TP-GT significantly reduced fluid volume during early resuscitation and possibly shortened ICU LOS. TP-GT may contribute to personalized shock management. However, further research should determine the best-performing tissue-perfusion assessment methods and identify specific patient phenotypes that may benefit most from this approach.

## Authors' contributions

Tamás Tóth: conceptualization, project administration, methodology, formal analysis, writing – original draft;

Patricia Schneidereit: investigation;

Julia Hollosi: methodology, supervision, writing – review and editing;

Dávid Lackó: methodology, supervision, writing – review and editing;

Daniel Louis Albert: investigation;

Bence Szabó: methodology, formal analysis, writing – original draft;

Caner Turan: conceptualization; supervision; writing – original draft;

László Zubek: conceptualization; supervision; writing – original draft;

Péter Hegyi: project administration, writing – review and editing;

Zsolt Molnár: conceptualization; supervision; writing – original draft.

All authors certify that they have participated sufficiently in the work to take public responsibility for the content, including participation in the concept, design, analysis, writing or revision of the manuscript.

## Consent for publication

Not applicable.

## Ethics approval and consent to participate

No ethical approval was required for this systematic review and meta-analysis, as all data were already published in peer-reviewed journals. No patients were involved in the design, conducting or interpretation of our study.

## Funding

Sponsors had no role in the design, data collection, analysis, interpretation, or manuscript preparation.

## Availability of data and material

The datasets used in this study can be found in the full-text articles or their supplements included in this systematic review and meta-analysis.

## Declaration of competing interest

None to declare.
