# Supplementary material for: The effectiveness of tissue-perfusion-guided resuscitation in shock: A systematic review and meta-analysis
Source: Ann Intensive Care. 2026 Jun 25;16:100106. doi: 10.1016/j.aicoj.2026.100106 (PMC13351399; doi:10.1016/j.aicoj.2026.100106)
Supplement: Supplementary file 1 [file mmc1.docx]

**SUPPLEMENTARY**

**The effectiveness of tissue-perfusion-guided resuscitation in shock
A systematic review and meta-analysis**

**Authors**

Tamás Tóth^2,7^, Patricia Schneidereit^2^, Julia Hollosi^2^, Dávid Lackó^2,8^, Bence Szabó^2^, Daniel Louis Albert^2^, Caner Turan^2,5^, László Zubek^2,5^, Péter Hegyi^1,2,3^, Zsolt Molnar^2,5,6^

**Affiliations:**

1. Institute for Translational Medicine, Medical School, University of Pécs, Pécs, Hungary
2. Centre for Translational Medicine, Semmelweis University, Budapest, Hungary
3. Institute of Pancreatic Diseases, Semmelweis University, Budapest, Hungary
4. Division of Gastroenterology, First Department of Medicine, Medical School, University of Pécs, Pécs, Hungary
5. Department of Anesthesiology and Intensive Therapy, Semmelweis University, Budapest, Hungary
6. Department of Anesthesiology and Intensive Therapy, Poznan University of Medical Sciences, Poznan, Poland
7. Department of Anaesthesiology and Intensive Therapy, Bajcsy-Zsilinszky Hospital, Budapest, Hungary.
8. Department of Interventional Radiology, Heart and Vascular Centre, Semmelweis University, Hungary

**Corresponding author**

Zsolt Molnár

E-mail address: [zsoltmolna@gmail.com](mailto:zsoltmolna@gmail.com)

**List of Tables:**

**Table S1.** Database-specific search strategies

**List of Figures:**

**Figure S1.** Funnel plot of comparison: TP-GT versus standard care, outcome: 30-day mortality.

**Figure S2.** Forest plot comparing 90-day mortality between TP-GT and standard care.

**Figure S3.** Funnel plot of comparison: TP-GT versus standard care, outcome: 90-day mortality.

**Figure S4.** Forest plot of sensitivity analysis (excluding ANDROMEDA-SHOCK-2) comparing 30-day mortality between TP-GT and standard care.

**Figure S5.** Funnel plot of comparison: TP-GT versus standard care (sensitivity analysis excluding ANDROMEDA-SHOCK-2), outcome: 30-day mortality.

**Figure S6.** Forest plot of sensitivity analysis (excluding DAMIS trial) comparing 30-day mortality between TP-GT and standard care in septic shock population.

**Figure S7.** Funnel plot of comparison: TP-GT versus standard care in septic shock population (sensitivity analysis excluding DAMIS trial), outcome: 30-day mortality.

**Figure S8.** Forest plot of subgroup analysis (CRT-guided intervention) comparing 30-day mortality between TP-GT and standard care.

**Figure S9.** Funnel plot of comparison: TP-GT versus standard care (CRT-guided intervention subgroup), outcome: 30-day mortality.

**Figure S10.** Funnel plot of comparison: TP-GT versus standard care, outcome: ICU length of stay.

**Figure S11.** Forest plot comparing hospital length of stay between TP-GT and standard care.

**Figure S12.** Funnel plot of comparison: TP-GT versus standard care, outcome: Hospital length of stay

**Figure S13.** Forest plot of sensitivity analysis (excluding ANDROMEDA-SHOCK-2) comparing ICU length of stay between TP-GT and standard care.

**Figure S14.** Funnel plot of comparison: TP-GT versus standard care (sensitivity analysis excluding ANDROMEDA-SHOCK-2), outcome: ICU length of stay.

**Figure S15.** Forest plot of sensitivity analysis (excluding DAMIS trial) comparing ICU length of stay between TP-GT and standard care in septic shock population.

**Figure S16.** Funnel plot of comparison: TP-GT versus standard care in septic shock population (sensitivity analysis excluding DAMIS trial), outcome: ICU length of stay.

**Figure S17.** Forest plot of subgroup analysis (CRT-guided intervention) comparing ICU length of stay between TP-GT and standard care.

**Figure S18.** Funnel plot of comparison: TP-GT versus standard care (CRT-guided intervention subgroup), outcome: ICU length of stay.

**Figure S19.** Funnel plot of comparison: TP-GT versus standard care, outcome: total fluid administration during the first 6 to 8 hours of resuscitation.

**Figure S20.** Forest plot comparing 24-hour fluid balance between TP-GT and standard care.

**Figure S21.** Funnel plot of comparison: TP-GT versus standard care, outcome: 24-hour fluid balance**.**

**Figure S22.** Forest plot of sensitivity analysis (excluding ANDROMEDA-SHOCK-2) comparing total fluid administration during the first 6 to 8 hours of resuscitation between TP-GT and standard care.

**Figure S23.** Funnel plot of comparison: TP-GT versus standard care (sensitivity analysis excluding ANDROMEDA-SHOCK-2), outcome: total fluid administration during the first 6 to 8 hours of resuscitation.

**Figure S24.** Forest plot of subgroup analysis (CRT-guided intervention) comparing total fluid administration during the first 6 to 8 hours of resuscitation between TP-GT and standard care.

**Figure S25.** Funnel plot of comparison: TP-GT versus standard care (CRT-guided intervention subgroup), outcome: total fluid administration during the first 6 to 8 hours of resuscitation.

**Figure S26.** Forest plot comparing vasopressor-free days between TP-GT and standard care.

**Figure S27.** Funnel plot of comparison: TP-GT versus standard care in critically ill patients, outcome: vasopressor-free days.

**Figure S28.** Forest plot comparing mechanical ventilation-free days between TP-GT and standard care.

**Figure S29.** Funnel plot of comparison: TP-GT versus standard care, outcome: mechanical ventilation-free days.

**Figure S30.** Forest plot comparing the need for renal replacement therapy between TP-GT and standard care.

**Figure S31.** Funnel plot of comparison: TP-GT versus standard care, outcome: need for renal replacement therapy.

**Figure S32.** Forest plot comparing the change in Sequential Organ Failure Assessment (SOFA) score at 72 hours between TP-GT and standard care.

**Figure S33.** Funnel plot of comparison: TP-GT versus standard care, outcome: change in Sequential Organ Failure Assessment (SOFA) score at 72 hours.

**Figure S34.** Risk of Bias assessment for 30-day mortality.

**Figure S35.** Risk of Bias assessment for 90-day mortality.

**Figure S36.** Risk of Bias assessment for intensive care unit length of stay.

**Figure S37.** Risk of Bias assessment for hospital length of stay.

**Figure S38.** Risk of Bias assessment for total fluid administered until the end of the 6–8 hour treatment period.

**Figure S39.** Risk of Bias assessment for fluid balance until the end of the 24-hour treatment period.

**Figure S40.** Risk of Bias assessment for vasopressor-free days.

**Figure S41.** Risk of Bias assessment for mechanical ventilation-free days.

**Figure S42.** Risk of Bias assessment for need for renal replacement therapy.

**Figure S43.** Risk of Bias assessment for 72h SOFA change.

**Figure S44.** GRADE assessment.

**Figure S45.** PRISMA 2020 checklist

**Figure S46.** Clinical implications of tissue-perfusion-guided therapy (TP-GT)visualized using the Academia Europaea ring diagram model.

**Table S1.** Database-specific search strategies

| **Pubmed** | ("shock" OR "resuscitation" OR (("haemodynamic" OR "hemodynamic") AND "instability")) AND (("intravital" AND "microscopy") OR "microcirculation" OR ("laser" AND "doppler" AND "flowmetry") OR "LDF" OR ("nailfold" AND "videocapillaroscopy") OR ("near-infrared" AND "spectroscopy") OR ("capillary" AND "refill" AND "time") OR "CRT" OR (("perfusion" OR ("microvascular" AND "flow")) AND "index") OR ("sidestream" AND "dark-field") OR "SDF" OR ("proportion" AND "perfused" AND "vessels") OR "PPV" OR "POEM" OR (("Backer" OR "mottling") AND "score") OR ("orthogonal" AND "polarization" AND "spectral")) AND (random* OR blind* OR controlled clinical trial [pt] OR randomized controlled trial [pt]) |
| --- | --- |
| **Embase** | (shock OR resuscitation OR ((haemodynamic OR hemodynamic) AND instability)) AND ((intravital AND microscopy) OR microcirculation OR (laser AND doppler AND flowmetry) OR LDF OR (nailfold AND videocapillaroscopy) OR (near-infrared AND spectroscopy) OR (capillary AND refill AND time) OR CRT OR ((perfusion OR (microvascular AND flow)) AND index) OR (sidestream AND dark-field) OR SDF OR (proportion AND perfused AND vessels) OR PPV OR POEM OR ((Backer OR mottling) AND score) OR (orthogonal AND polarization AND spectral)) AND (random* OR blind* OR 'randomized controlled trial'/exp OR 'controlled clinical trial'/de) |
| **CENTRAL (Cochrane)** | (shock OR resuscitation OR ((haemodynamic OR hemodynamic) AND instability)) AND ((intravital AND microscopy) OR microcirculation OR (laser AND doppler AND flowmetry) OR LDF OR (nailfold AND videocapillaroscopy) OR ("near-infrared" AND spectroscopy) OR (capillary AND refill AND time) OR CRT OR ((perfusion OR (microvascular AND flow)) AND index) OR (sidestream AND "dark-field") OR SDF OR (proportion AND perfused AND vessels) OR PPV OR POEM OR ((Backer OR mottling) AND score) OR (orthogonal AND polarization AND spectral)) |

**Figure S1.** Funnel plot of comparison: TP-GT versus standard care, outcome: 30-day mortality**.
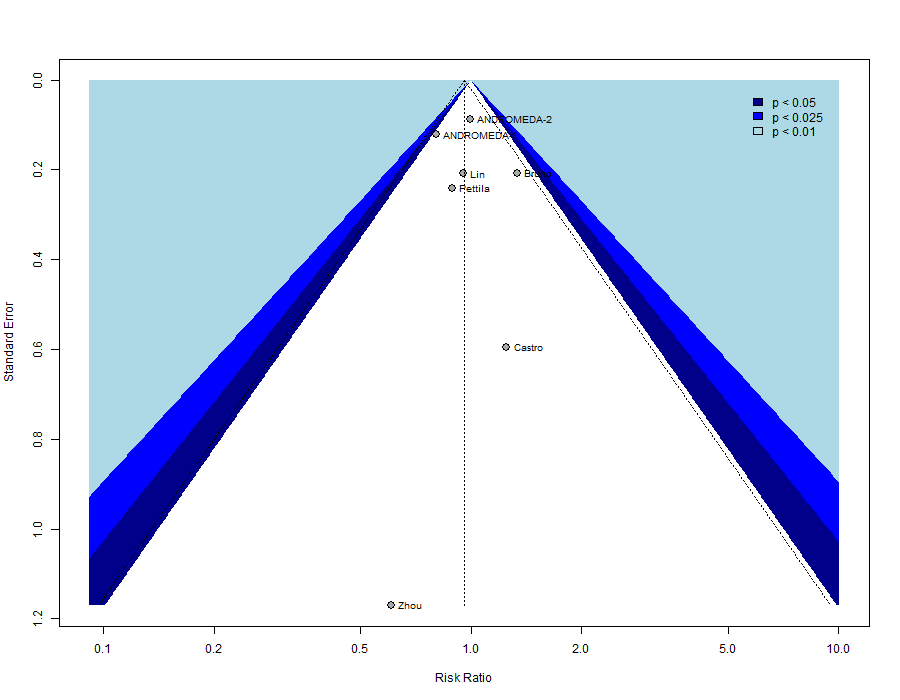
**

**Figure S2.** Forest plot comparing 90-day mortality between TP-GT and standard care.


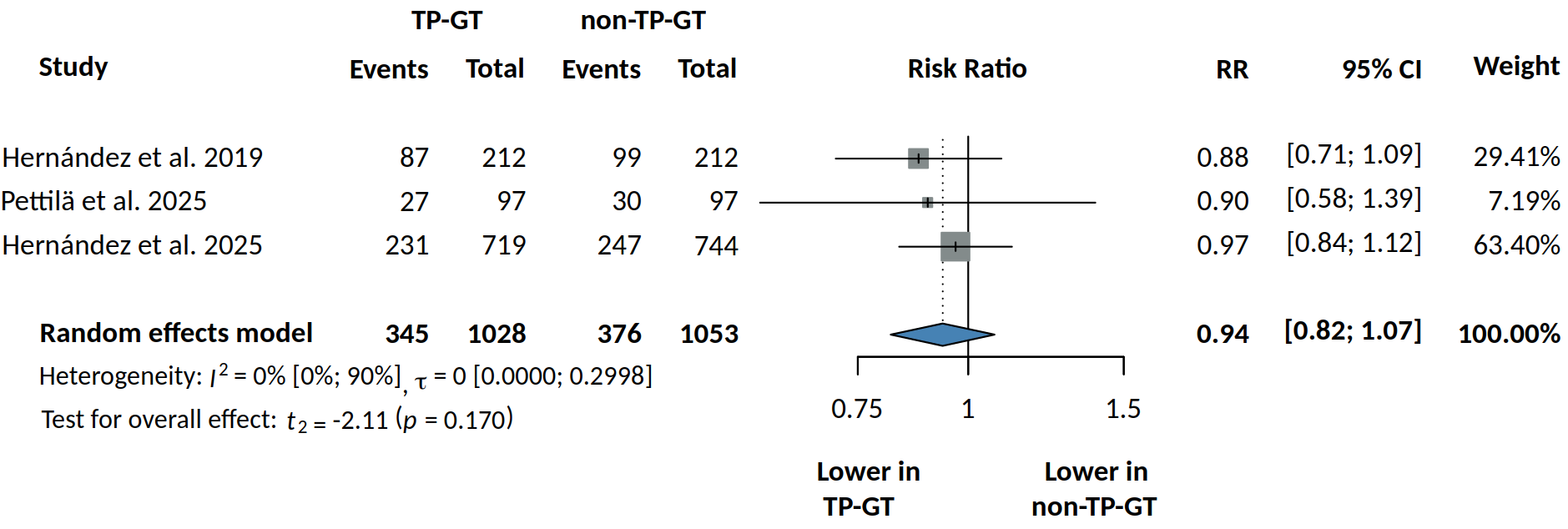


*The size of the squares is proportional to the weight of each study in the meta-analysis. The horizontal lines represent 95% CIs. The diamond represents the overall pooled effect, calculated using a Mantel-Haenszel random-effects model. Statistical heterogeneity was assessed using the I^2^ statistic test. CI = confidence interval; RR = risk ratio*

**Figure S3.** Funnel plot of comparison: TP-GT versus standard care, outcome: 90-day mortality.

*
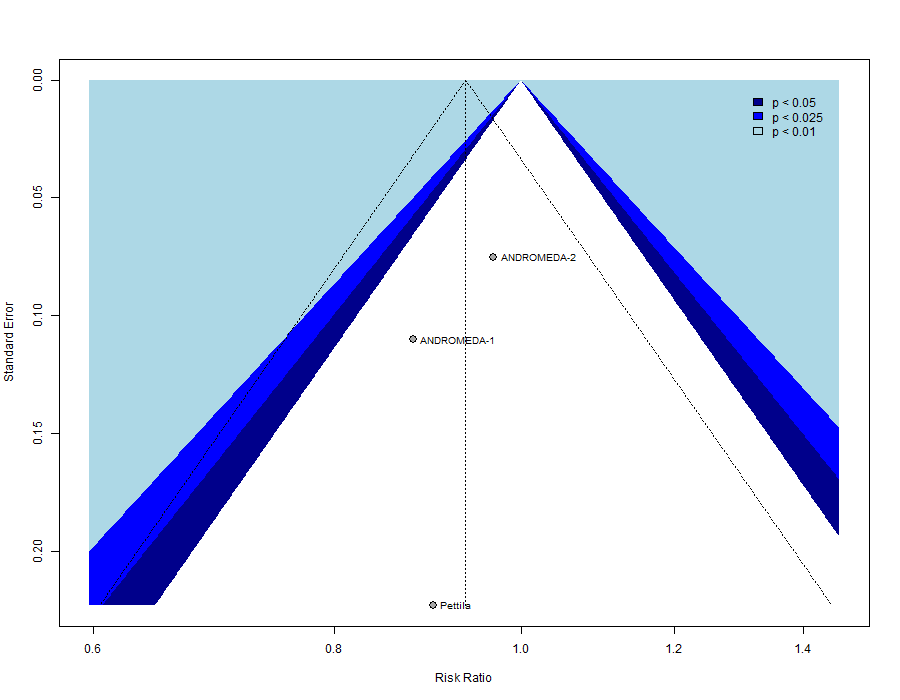
*

**Figure S4.** Forest plot of sensitivity analysis (excluding ANDROMEDA-SHOCK-2) comparing 30-day mortality between TP-GT and standard care.

**
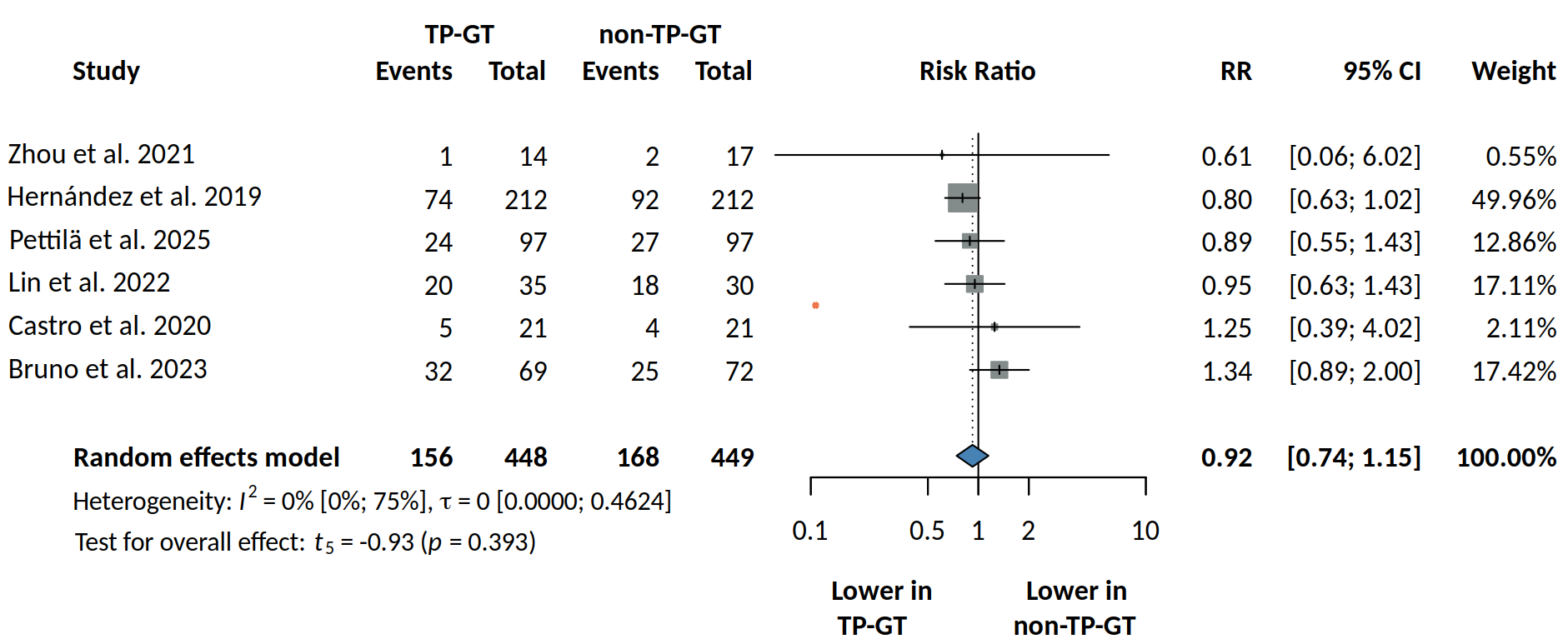
**

*The size of the squares is proportional to the weight of each study in the meta-analysis. The horizontal lines represent 95% CIs. The diamond represents the overall pooled effect, calculated using a Mantel-Haenszel random-effects model. Statistical heterogeneity was assessed using the I^2^ statistic test. CI = confidence interval; RR = risk ratio*

**Figure S5.** Funnel plot of comparison: TP-GT versus standard care (sensitivity analysis excluding ANDROMEDA-SHOCK-2), outcome: 30-day mortality.

**
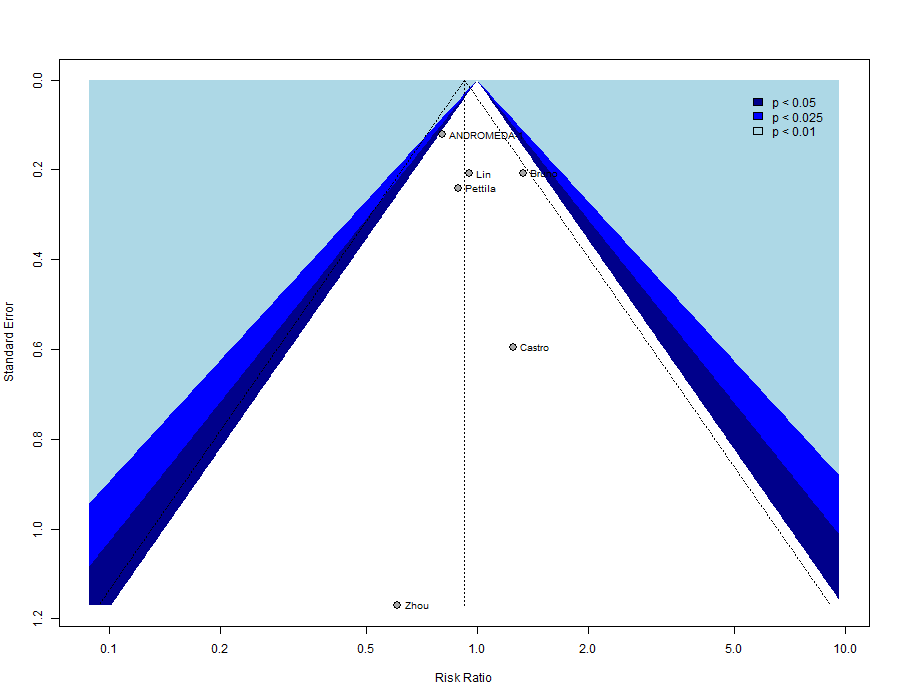
**

**Figure S6.** Forest plot of sensitivity analysis (excluding DAMIS trial) comparing 30-day mortality between TP-GT and standard care in septic shock population.

**
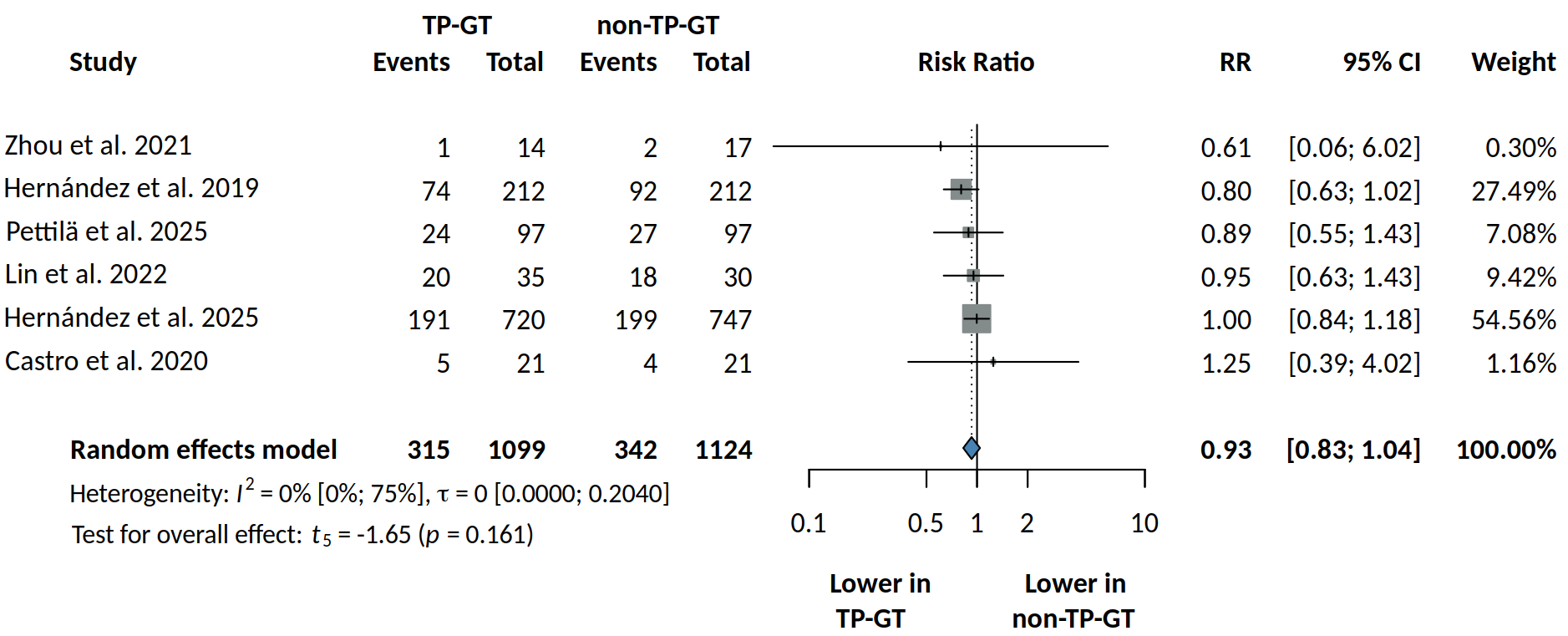
**

*The size of the squares is proportional to the weight of each study in the meta-analysis. The horizontal lines represent 95% CIs. The diamond represents the overall pooled effect, calculated using a Mantel-Haenszel random-effects model. Statistical heterogeneity was assessed using the I^2^ statistic test. CI = confidence interval; RR = risk ratio*

**Figure S7.** Funnel plot of comparison: TP-GT versus standard care in septic shock population (sensitivity analysis excluding DAMIS trial), outcome: 30-day mortality.

**
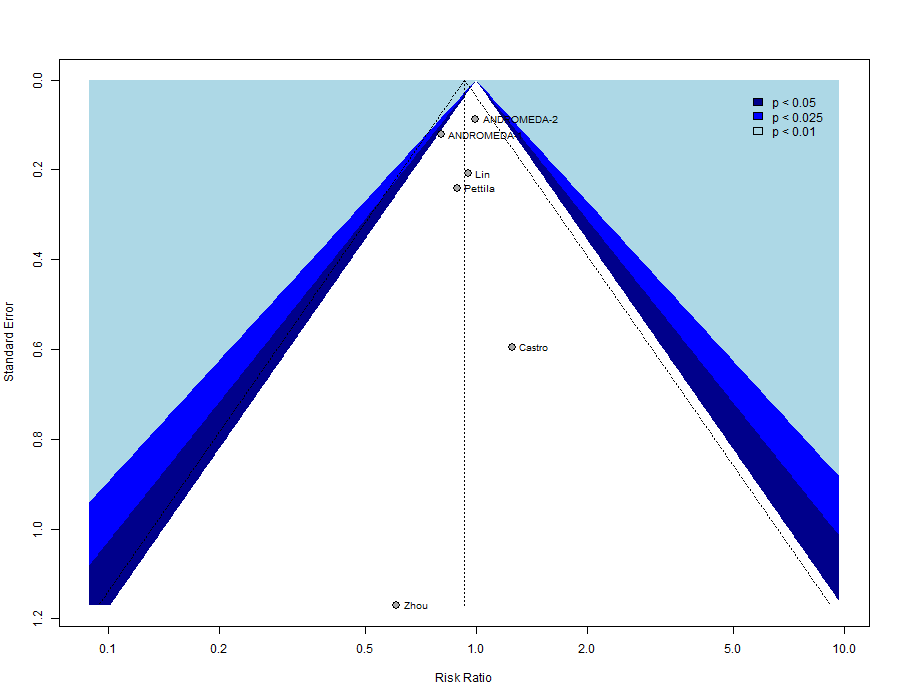
**

**Figure S8.** Forest plot of subgroup analysis (CRT-guided intervention) comparing 30-day mortality between TP-GT and standard care.

**
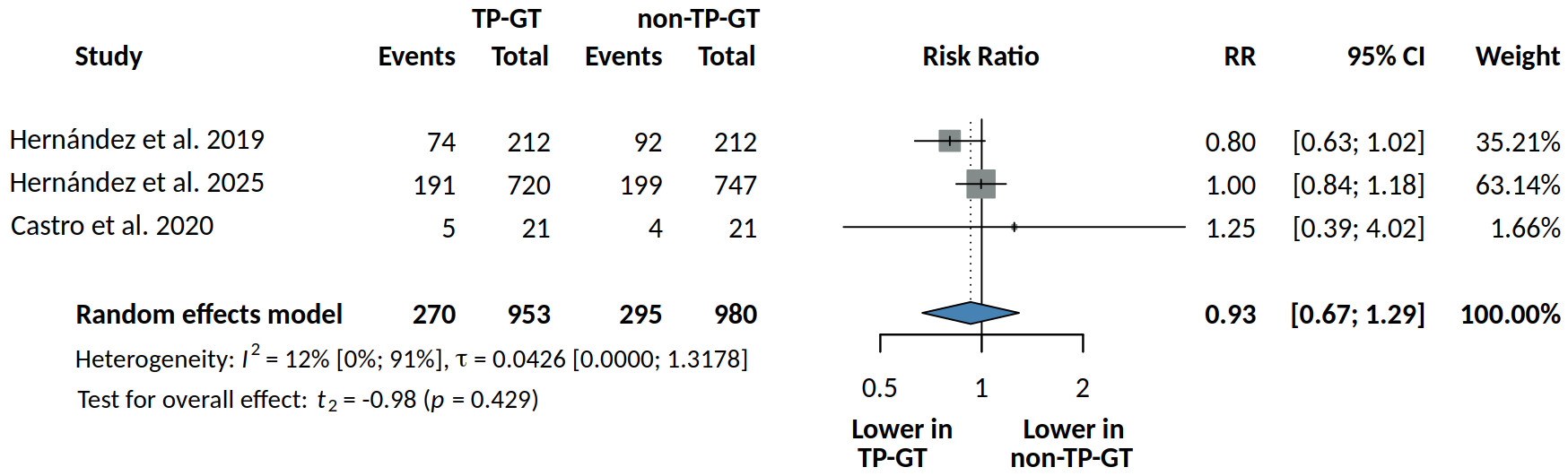
**

*The size of the squares is proportional to the weight of each study in the meta-analysis. The horizontal lines represent 95% CIs. The diamond represents the overall pooled effect, calculated using a Mantel-Haenszel random-effects model. Statistical heterogeneity was assessed using the I^2^ statistic test. CI = confidence interval; RR = risk ratio*

**Figure S9.** Funnel plot of comparison: TP-GT versus standard care (CRT-guided intervention subgroup), outcome: 30-day mortality.


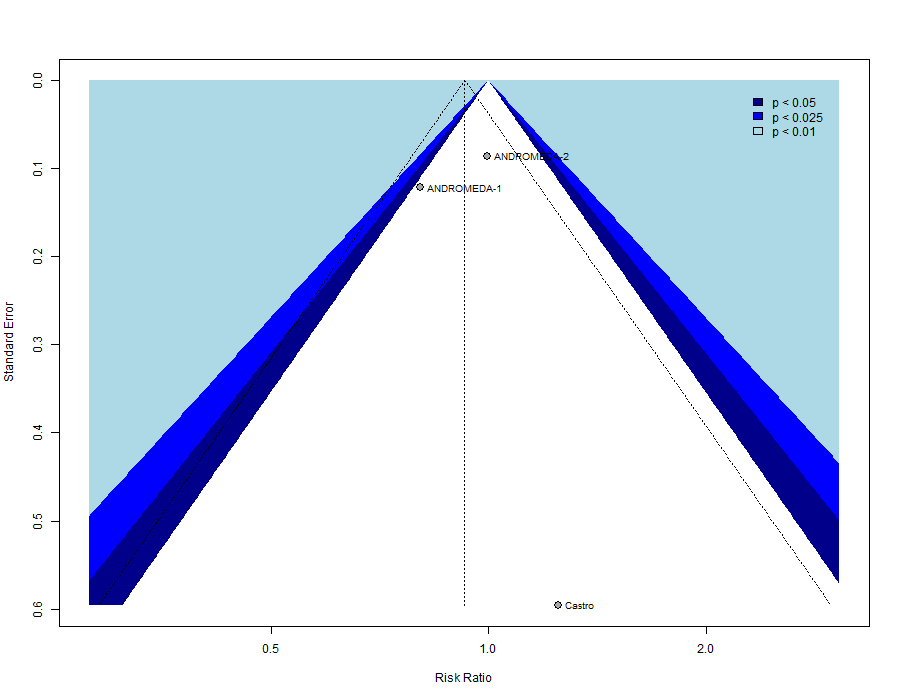


**Figure S10.** Funnel plot of comparison: TP-GT versus standard care, outcome: ICU length of stay.


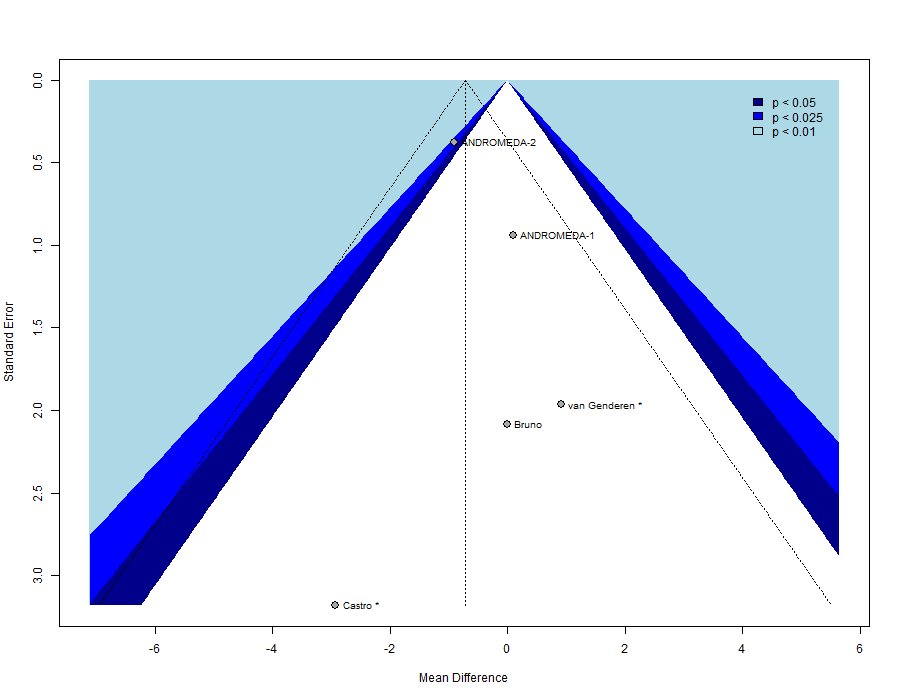


**Figure S11.** Forest plot comparing hospital length of stay between TP-GT and standard care.

**
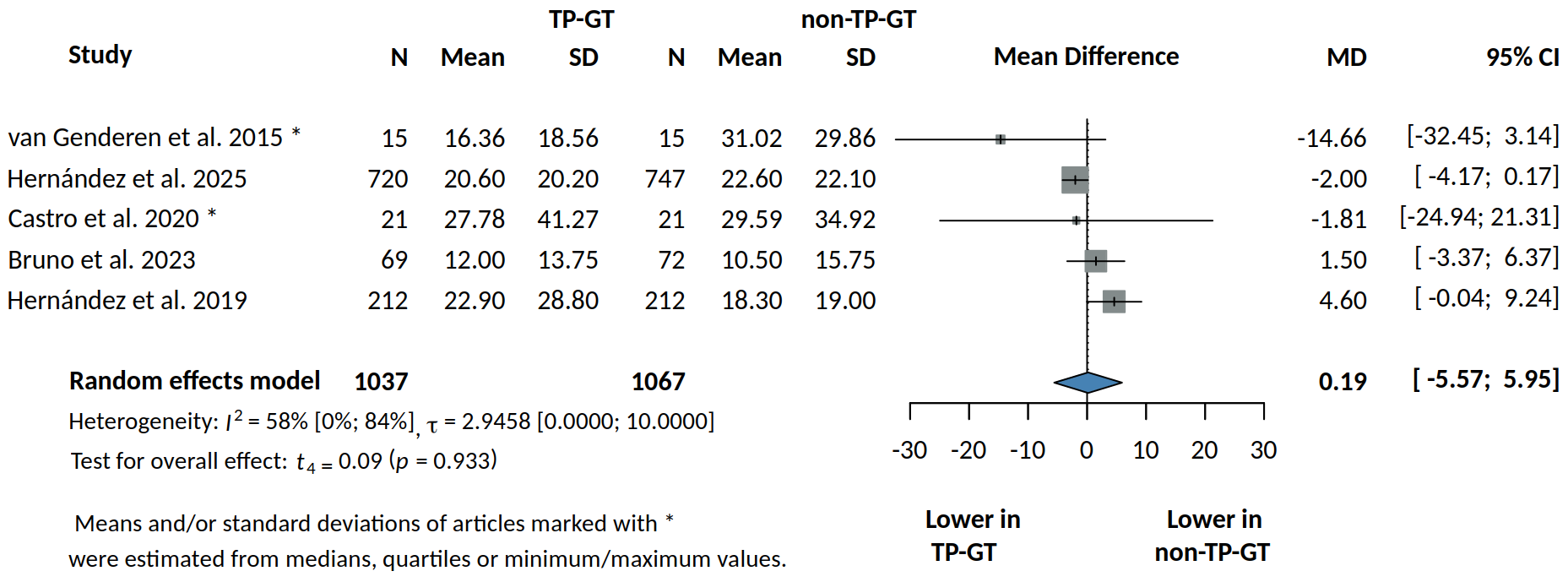
**

*The size of the squares is proportional to the weight of each study in the meta-analysis. The horizontal lines represent 95% CIs. The diamond represents the overall pooled effect, calculated using an inverse variance random-effects model. Means and/or standard deviations for select studies were estimated from medians, quartiles, or minimum/maximum values. Statistical heterogeneity was assessed using the I^2^ statistic test. CI = confidence interval; MD = mean difference.*

**Figure S12.** Funnel plot of comparison: TP-GT versus standard care, outcome: Hospital length of stay.


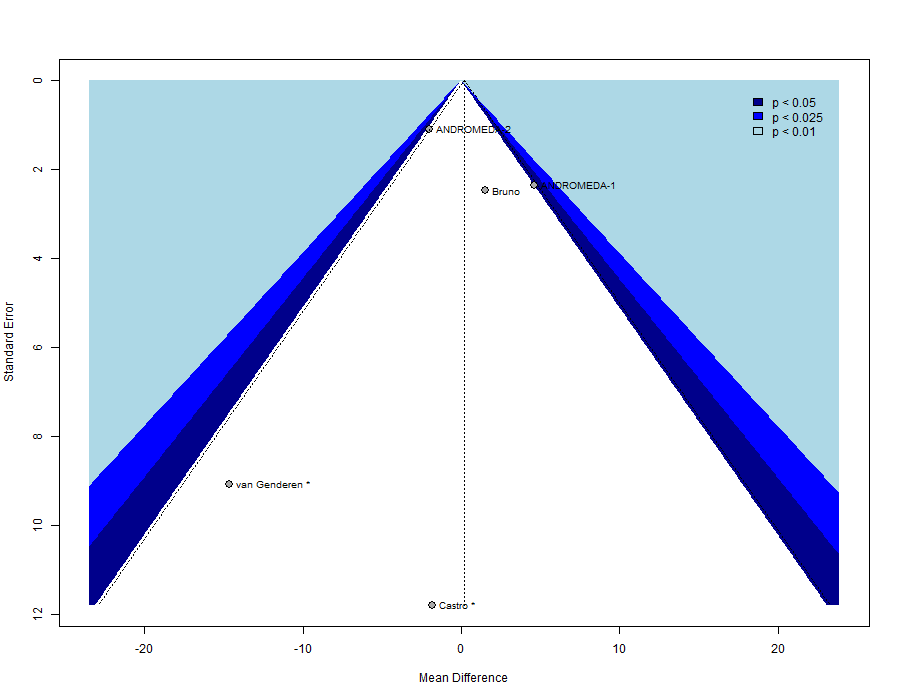


**Figure S13.** Forest plot of sensitivity analysis (excluding ANDROMEDA-SHOCK-2) comparing ICU length of stay between TP-GT and standard care.


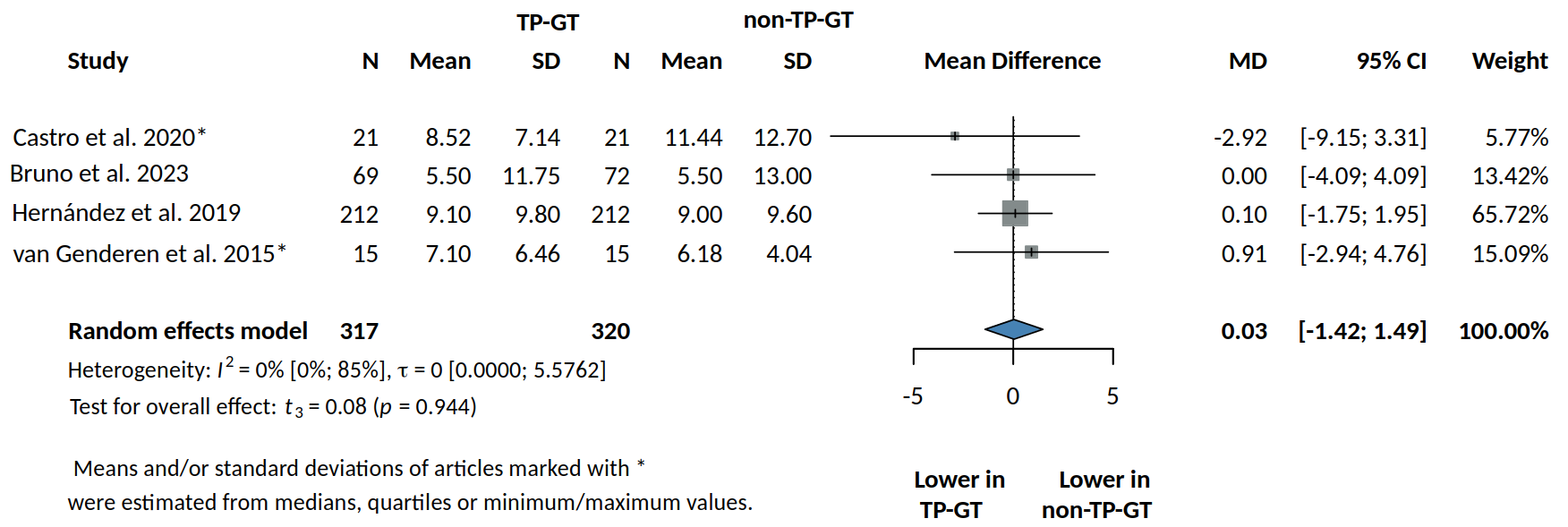


*The size of the squares is proportional to the weight of each study in the meta-analysis. The horizontal lines represent 95% CIs. The diamond represents the overall pooled effect, calculated using an inverse variance random-effects model. Means and/or standard deviations for select studies were estimated from medians, quartiles, or minimum/maximum values. Statistical heterogeneity was assessed using the I^2^ statistic test. CI = confidence interval; MD = mean difference.*

**Figure S14.** Funnel plot of comparison: TP-GT versus standard care (sensitivity analysis excluding ANDROMEDA-SHOCK-2), outcome: ICU length of stay.


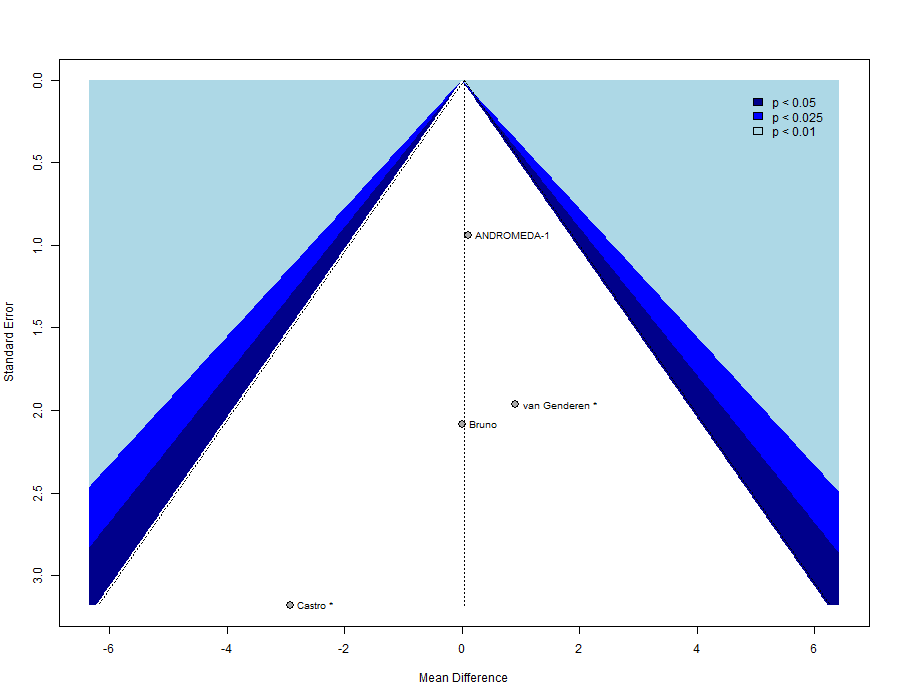


**Figure S15.** Forest plot of sensitivity analysis (excluding DAMIS trial) comparing ICU length of stay between TP-GT and standard care in septic shock population.

**
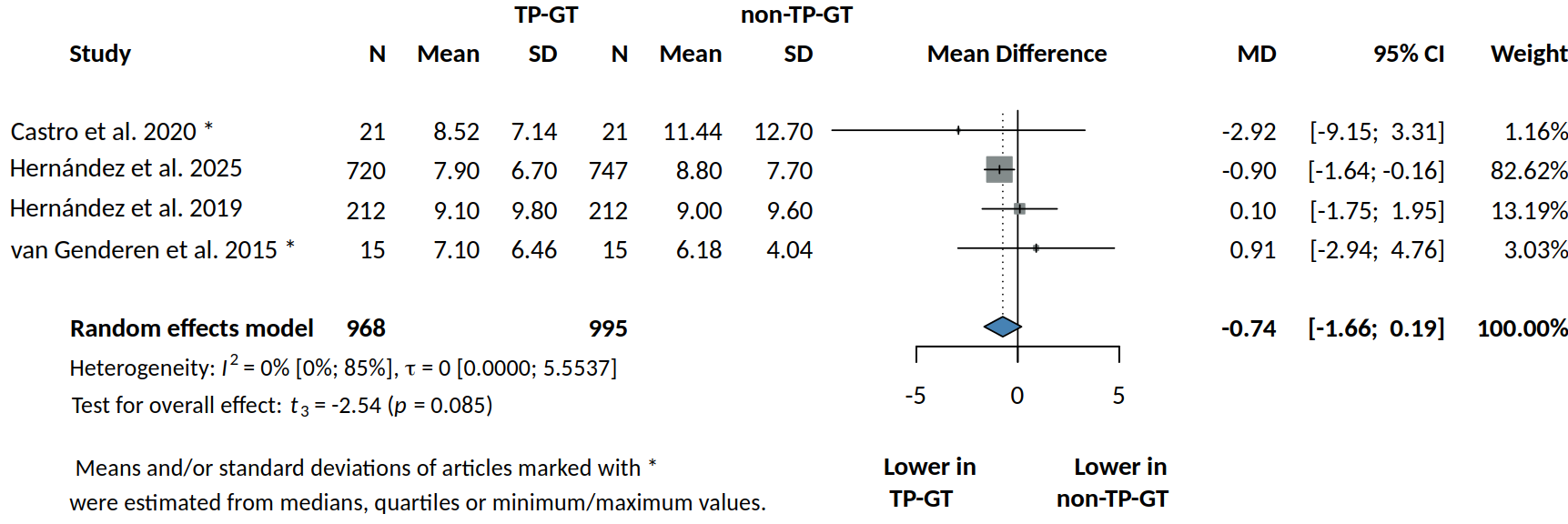
**

*The size of the squares is proportional to the weight of each study in the meta-analysis. The horizontal lines represent 95% CIs. The diamond represents the overall pooled effect, calculated using an inverse variance random-effects model. Means and/or standard deviations for select studies were estimated from medians, quartiles, or minimum/maximum values. Statistical heterogeneity was assessed using the I^2^ statistic test. CI = confidence interval; ICU = intensive care unit; MD = mean difference.*

**Figure S16.** Funnel plot of comparison: TP-GT versus standard care in septic shock population (sensitivity analysis excluding DAMIS trial), outcome: ICU length of stay.

**
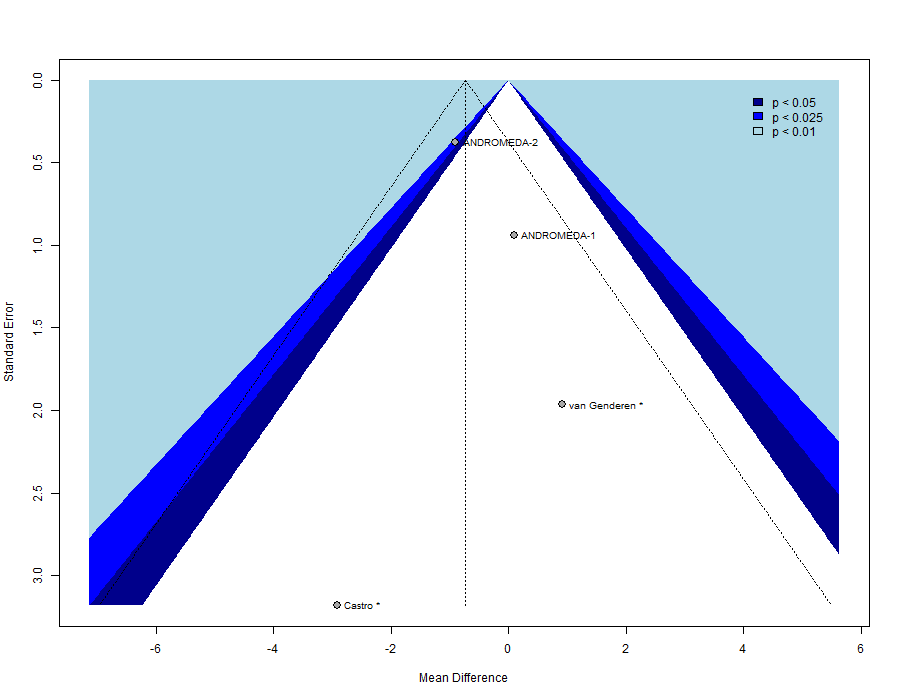
**

**Figure S17.** Forest plot of subgroup analysis (CRT-guided intervention) comparing ICU length of stay between TP-GT and standard care.


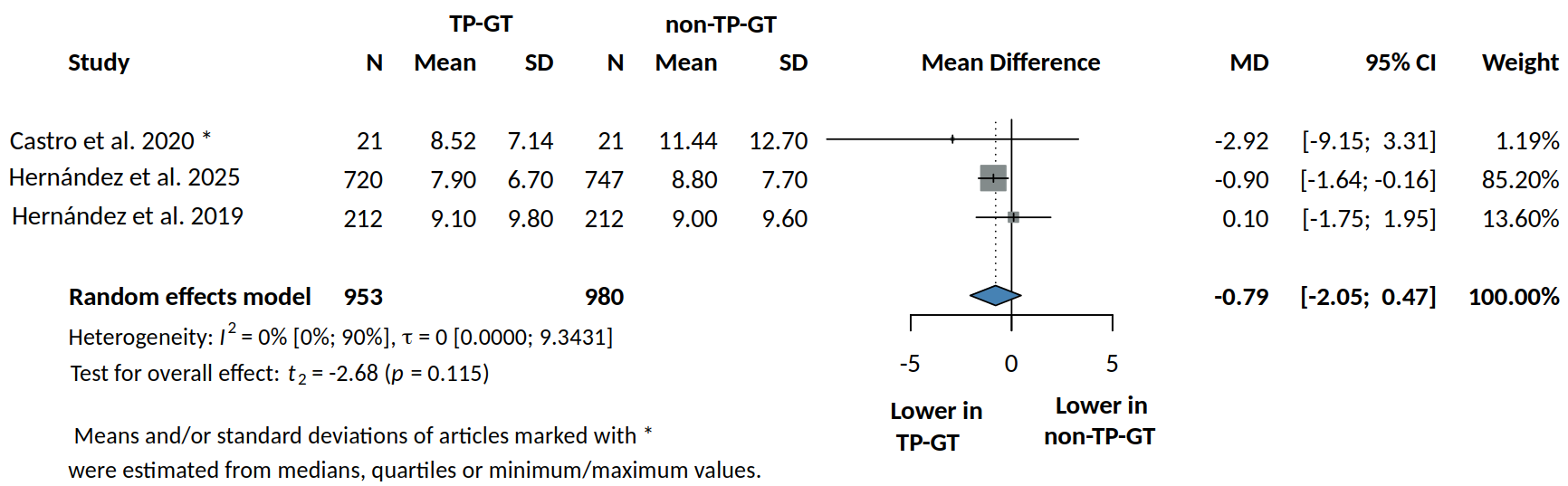


*The size of the squares is proportional to the weight of each study in the meta-analysis. The horizontal lines represent 95% CIs. The diamond represents the overall pooled effect, calculated using an inverse variance random-effects model. Means and/or standard deviations for select studies were estimated from medians, quartiles, or minimum/maximum values. Statistical heterogeneity was assessed using the I^2^ statistic test. CI = confidence interval; ICU = intensive care unit; MD = mean difference*

**Figure S18.** Funnel plot of comparison: TP-GT versus standard care (CRT-guided intervention subgroup), outcome: ICU length of stay.

**
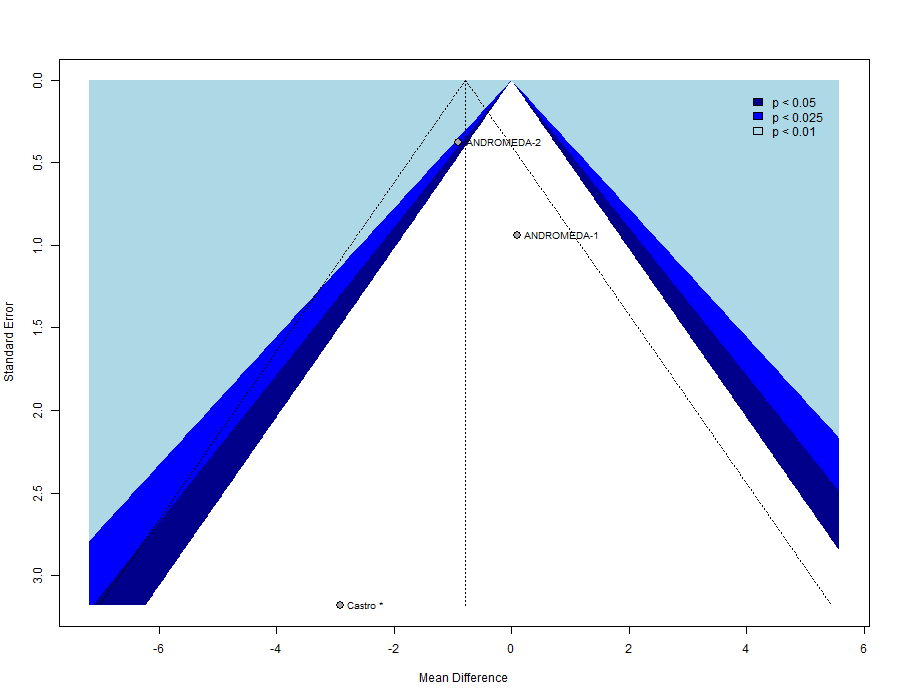
**

**Figure S19.** Funnel plot of comparison: TP-GT versus standard care, outcome: total fluid administration during the first 6 to 8 hours of resuscitation.

**
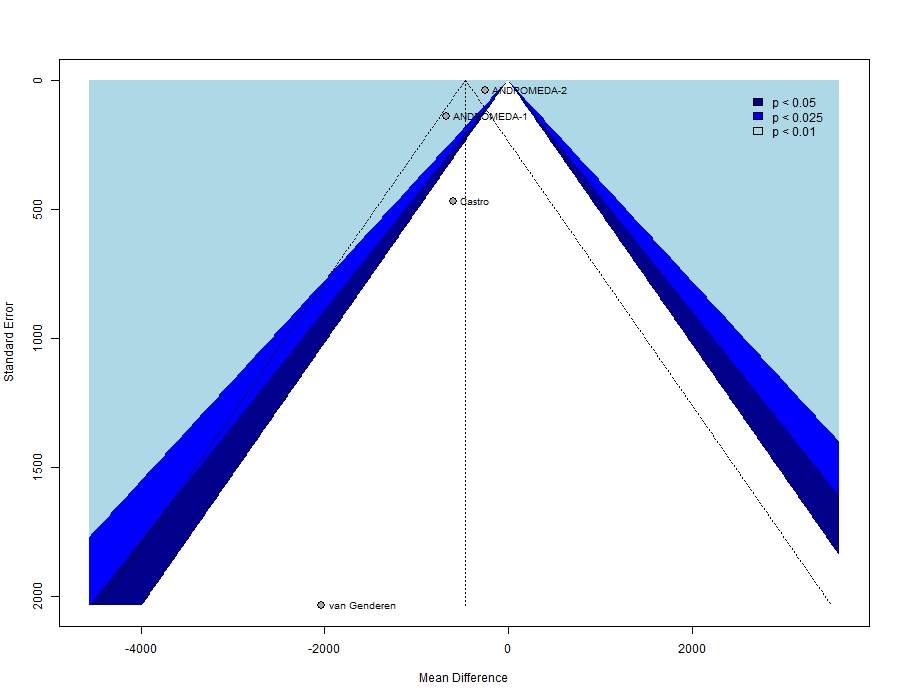
**

**Figure S20.** Forest plot comparing 24-hour fluid balance between TP-GT and standard care.

**
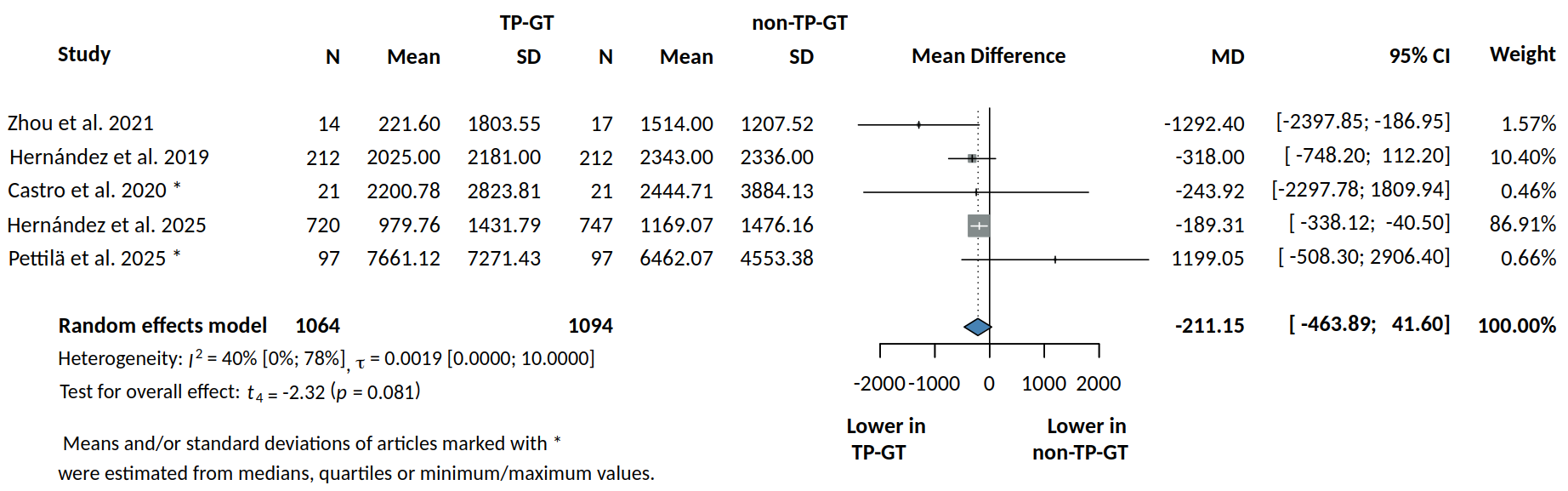
**

*The size of the squares is proportional to the weight of each study in the meta-analysis. The horizontal lines represent 95% CIs. The diamond represents the overall pooled effect, calculated using an inverse variance random-effects model. Means and/or standard deviations for select studies were estimated from medians, quartiles, or minimum/maximum values. Statistical heterogeneity was assessed using the I^2^ statistic test. CI = confidence interval; MD = mean difference.*

**Figure S21.** Funnel plot of comparison: TP-GT versus standard care, outcome: 24-hour fluid balance**.**

**
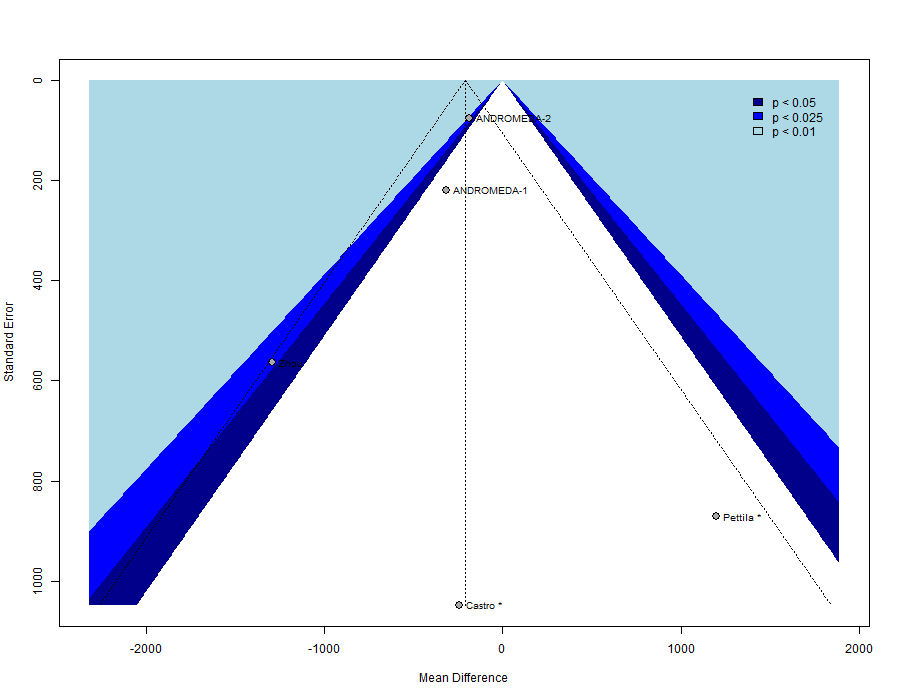
**

**Figure S22.** Forest plot of sensitivity analysis (excluding ANDROMEDA-SHOCK-2) comparing total fluid administration during the first 6 to 8 hours of resuscitation between TP-GT and standard care.

**
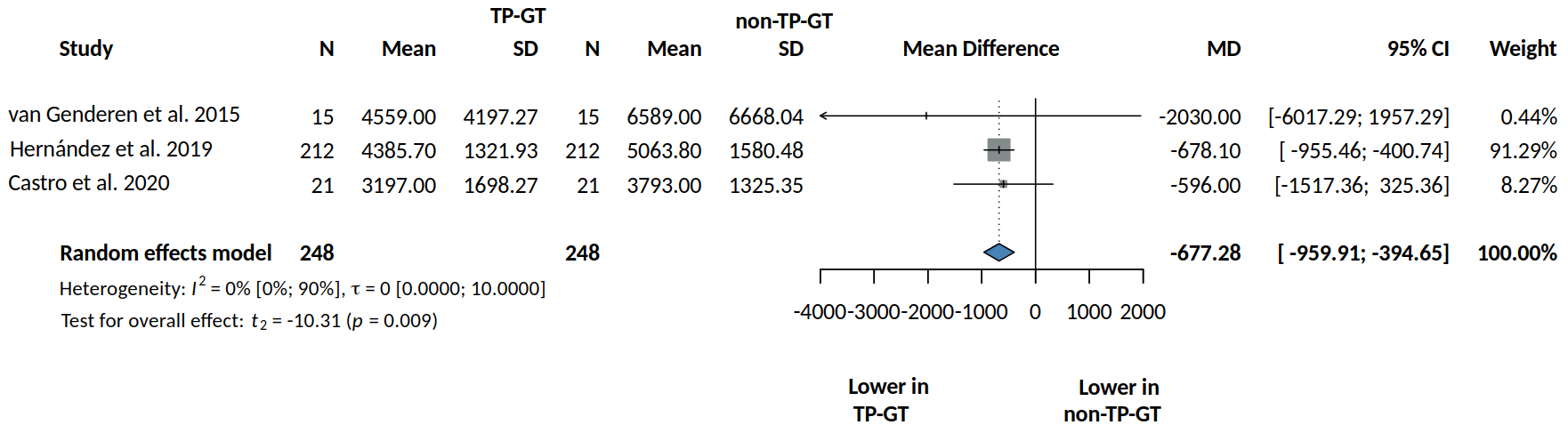
**

*The size of the squares is proportional to the weight of each study in the meta-analysis. The horizontal lines represent 95% CIs. The diamond represents the overall pooled effect, calculated using an inverse variance random-effects model. Statistical heterogeneity was assessed using the I^2^ statistic test. CI = confidence interval; MD = mean difference.*

**Figure S23.** Funnel plot of comparison: TP-GT versus standard care (sensitivity analysis excluding ANDROMEDA-SHOCK-2), outcome: total fluid administration during the first 6 to 8 hours of resuscitation.

**
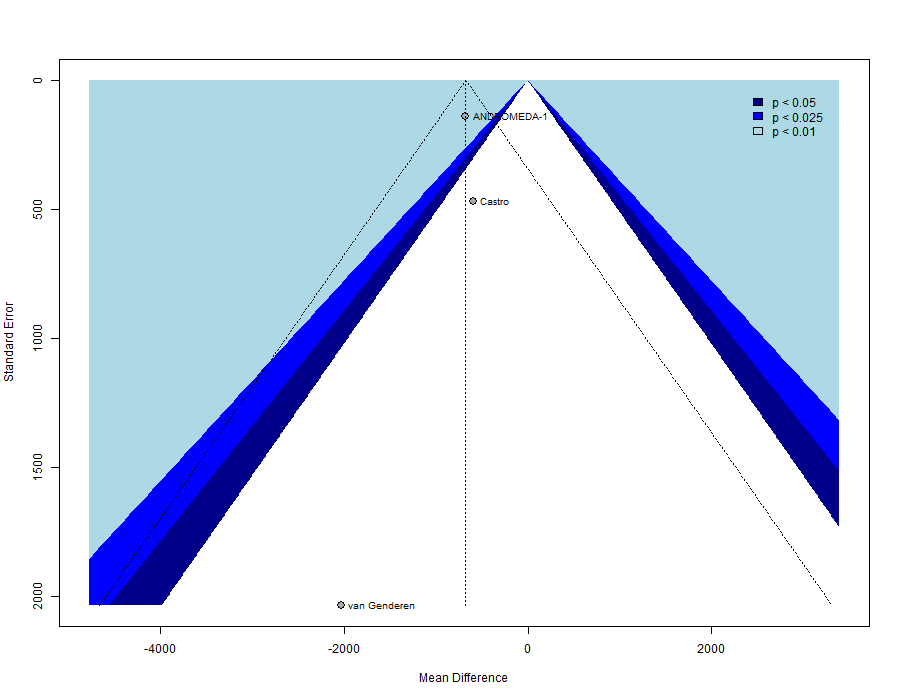
**

**Figure S24.** Forest plot of subgroup analysis (CRT-guided intervention) comparing total fluid administration during the first 6 to 8 hours of resuscitation between TP-GT and standard care.


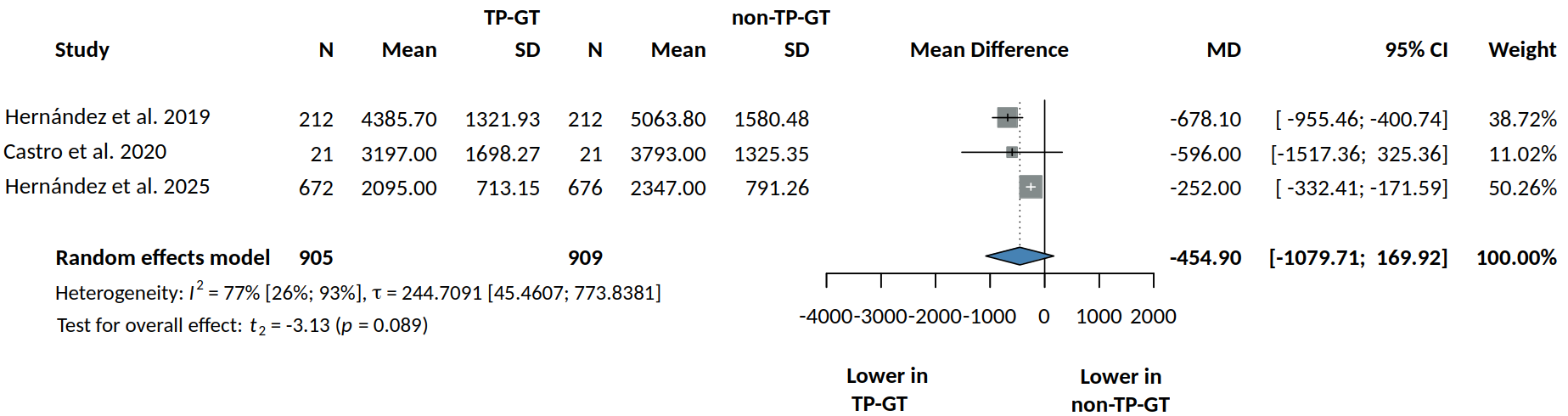


*The size of the squares is proportional to the weight of each study in the meta-analysis. The horizontal lines represent 95% CIs. The diamond represents the overall pooled effect, calculated using an inverse variance random-effects model. Statistical heterogeneity was assessed using the I^2^ statistic test. CI = confidence interval; MD = mean difference.*

**Figure S25.** Funnel plot of comparison: TP-GT versus standard care (CRT-guided intervention subgroup), outcome: total fluid administration during the first 6 to 8 hours of resuscitation.

**
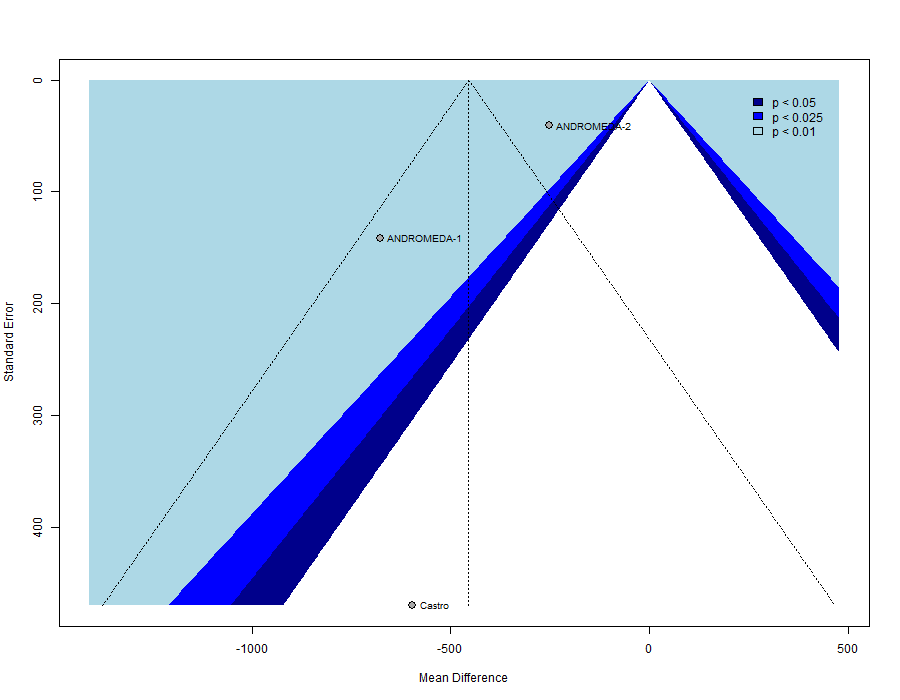
**

**Figure S26.** Forest plot comparing vasopressor-free days between TP-GT and standard care.


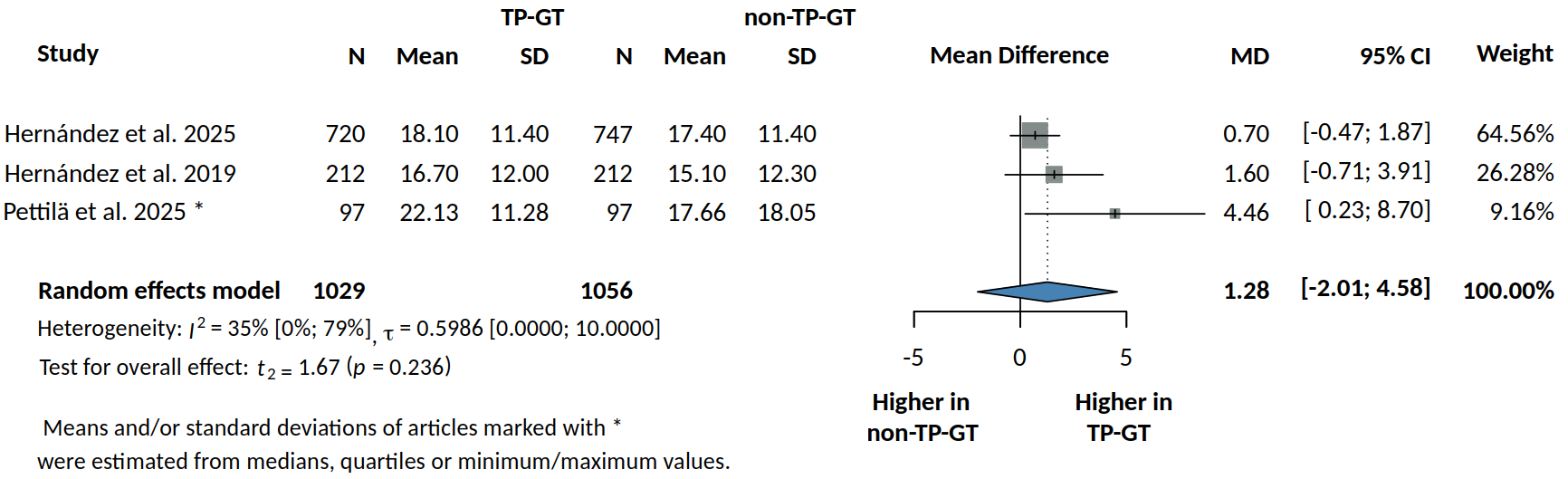


*The size of the squares is proportional to the weight of each study in the meta-analysis. The horizontal lines represent 95% CIs. The diamond represents the overall pooled effect, calculated using an inverse variance random-effects model. Means and/or standard deviations for select studies were estimated from medians, quartiles, or minimum/maximum values. Statistical heterogeneity was assessed using the I^2^ statistic test. CI = confidence interval; MD = mean difference.*

**Figure S27.** Funnel plot of comparison: TP-GT versus standard care in critically ill patients, outcome: vasopressor-free days.

**
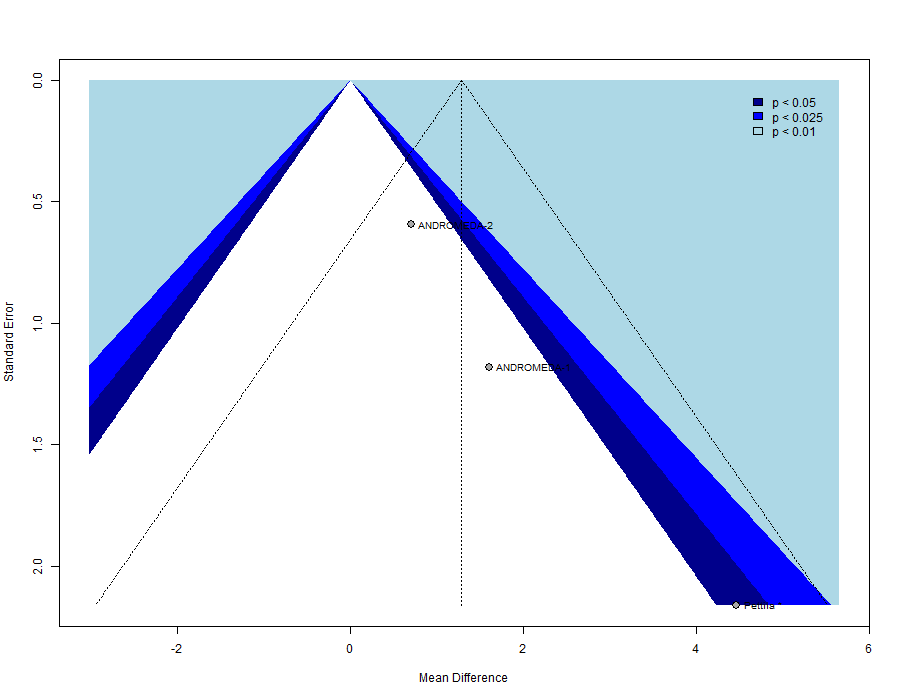
**

**Figure S28.** Forest plot comparing mechanical ventilation-free days between TP-GT and standard care.


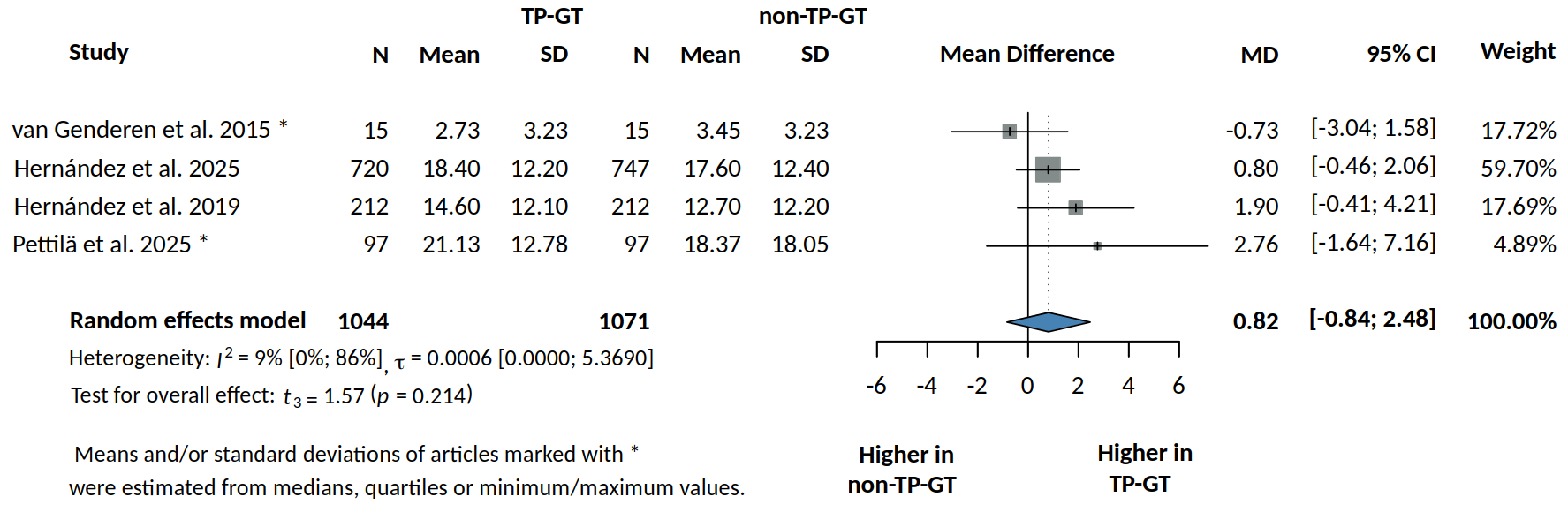


*The size of the squares is proportional to the weight of each study in the meta-analysis. The horizontal lines represent 95% CIs. The diamond represents the overall pooled effect, calculated using an inverse variance random-effects model. Means and/or standard deviations for select studies were estimated from medians, quartiles, or minimum/maximum values. Statistical heterogeneity was assessed using the I^2^ statistic test. CI = confidence interval; MD = mean difference.*

**Figure S29.** Funnel plot of comparison: TP-GT versus standard care, outcome: mechanical ventilation-free days.

**
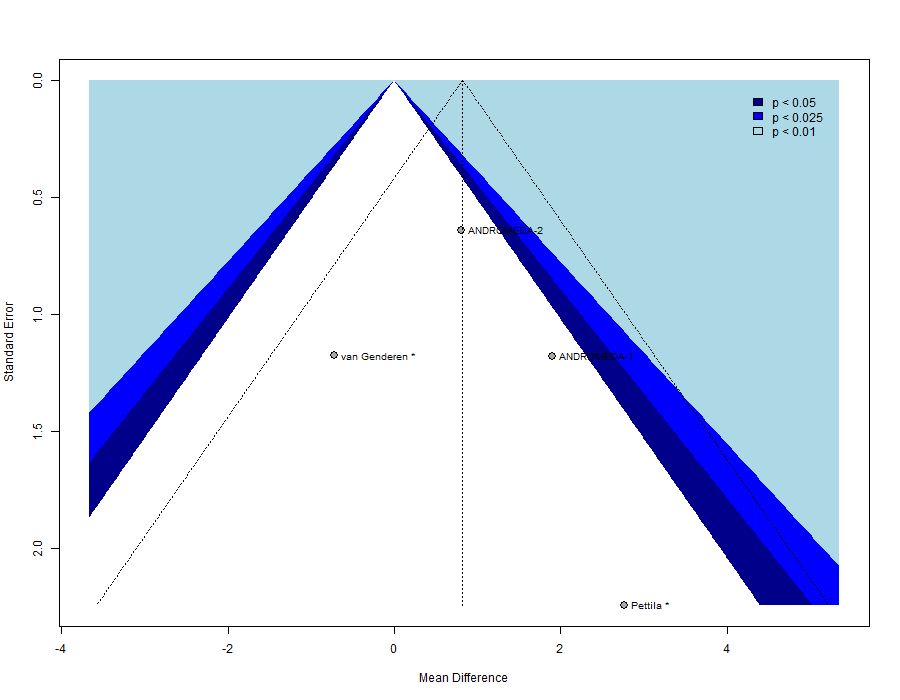
**

**Figure S30.** Forest plot comparing the need for renal replacement therapy between TP-GT and standard care.


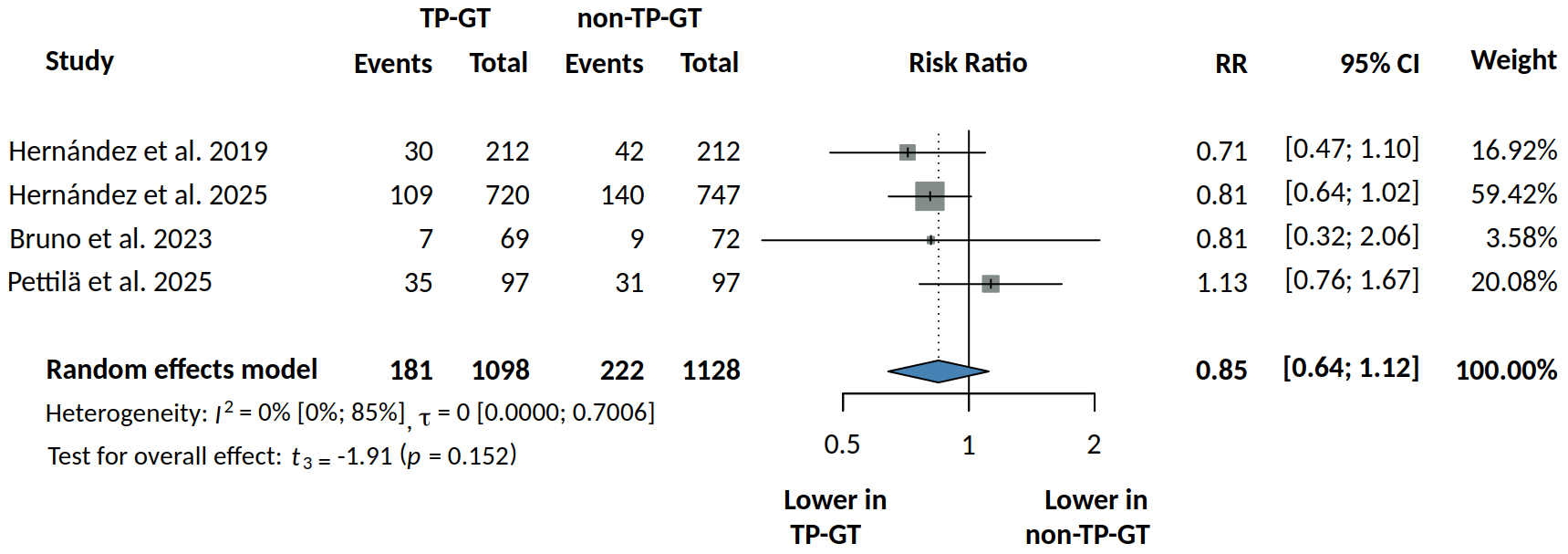


*The size of the squares is proportional to the weight of each study in the meta-analysis. The horizontal lines represent 95% CIs. The diamond represents the overall pooled effect, calculated using a Mantel-Haenszel random-effects model. Statistical heterogeneity was assessed using the I^2^ statistic test. CI = confidence interval; RR = risk ratio.*

**Figure S31.** Funnel plot of comparison: TP-GT versus standard care, outcome: need for renal replacement therapy.

**
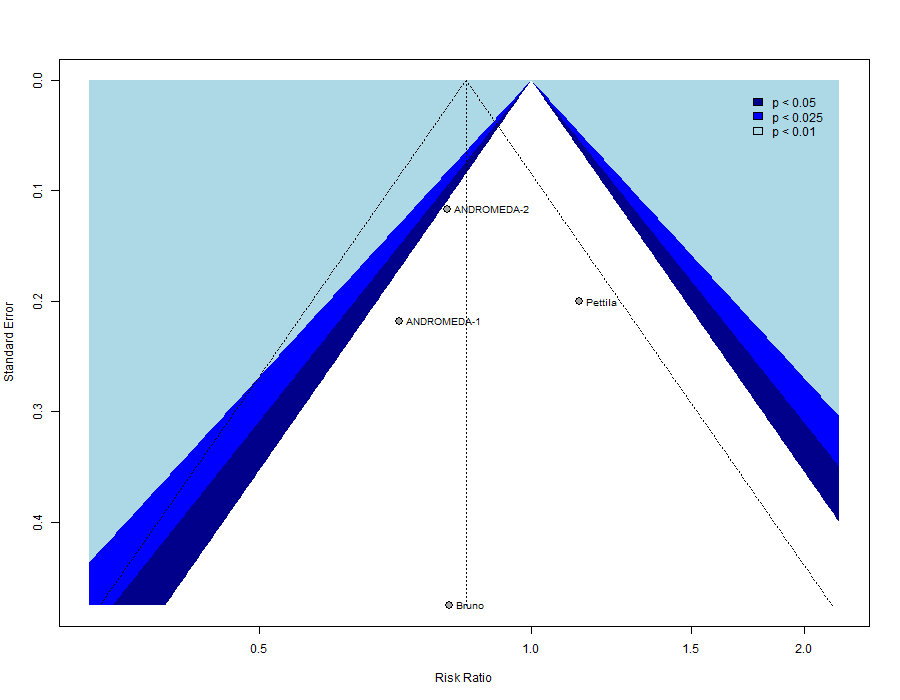
**

**Figure S32.** Forest plot comparing the change in Sequential Organ Failure Assessment (SOFA) score at 72 hours between TP-GT and standard care.


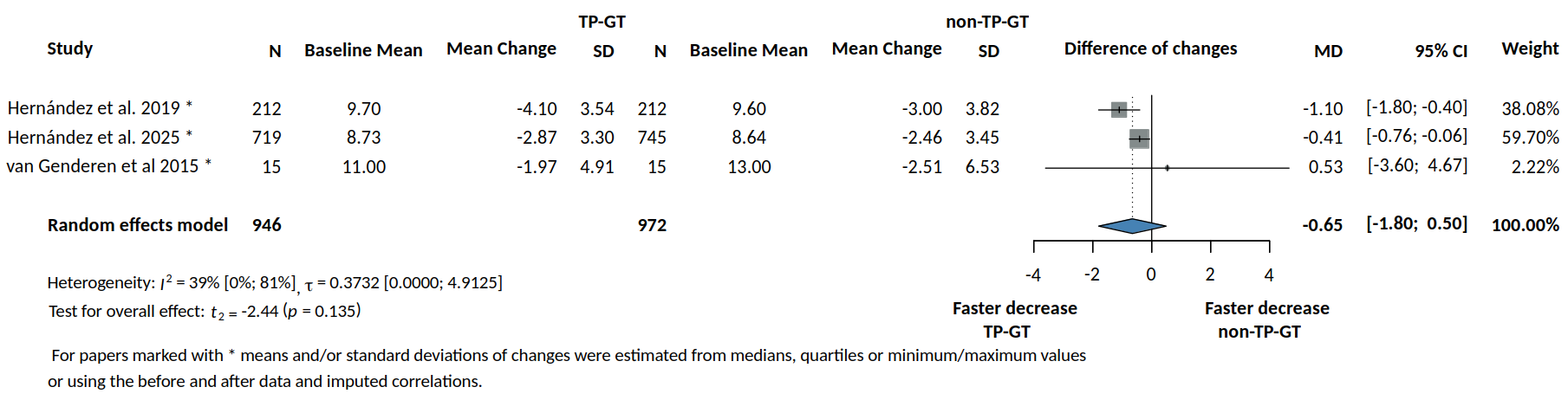


*The size of the squares is proportional to the weight of each study in the meta-analysis. The horizontal lines represent 95% CIs. The diamond represents the overall pooled effect, calculated using an inverse variance random-effects model. For select papers, means and/or standard deviations of changes were estimated from medians, quartiles, minimum/maximum values, or using before-and-after data with imputed correlations. Statistical heterogeneity was assessed using the I^2^ statistic test. CI = confidence interval; MD = mean difference.*

**Figure S33.** Funnel plot of comparison: TP-GT versus standard care, outcome: change in Sequential Organ Failure Assessment (SOFA) score at 72 hours.


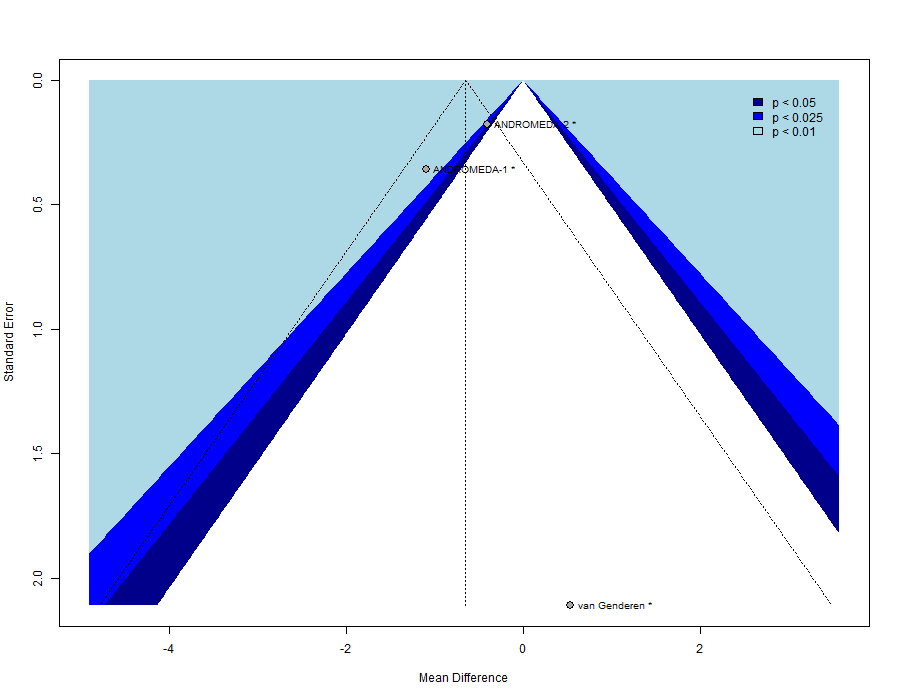


**Figure S34.** Risk of Bias assessment for 30-day mortality.

**
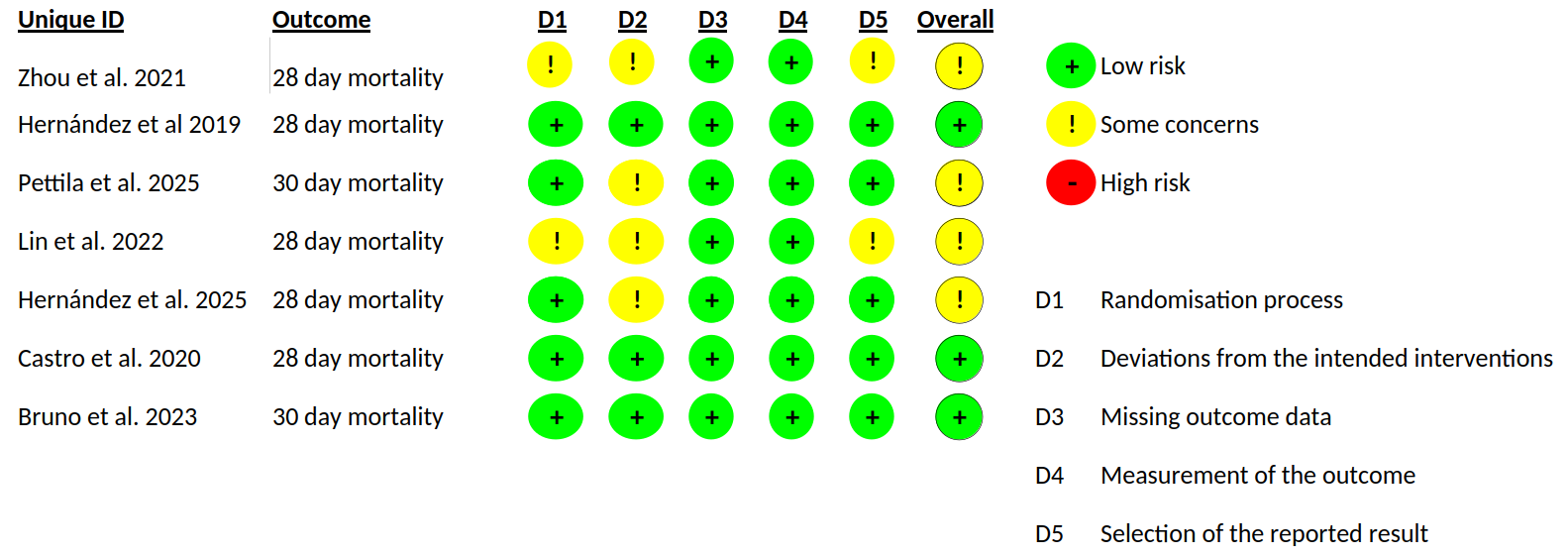
**

**Figure S35.** Risk of Bias assessment for 90-day mortality.

**
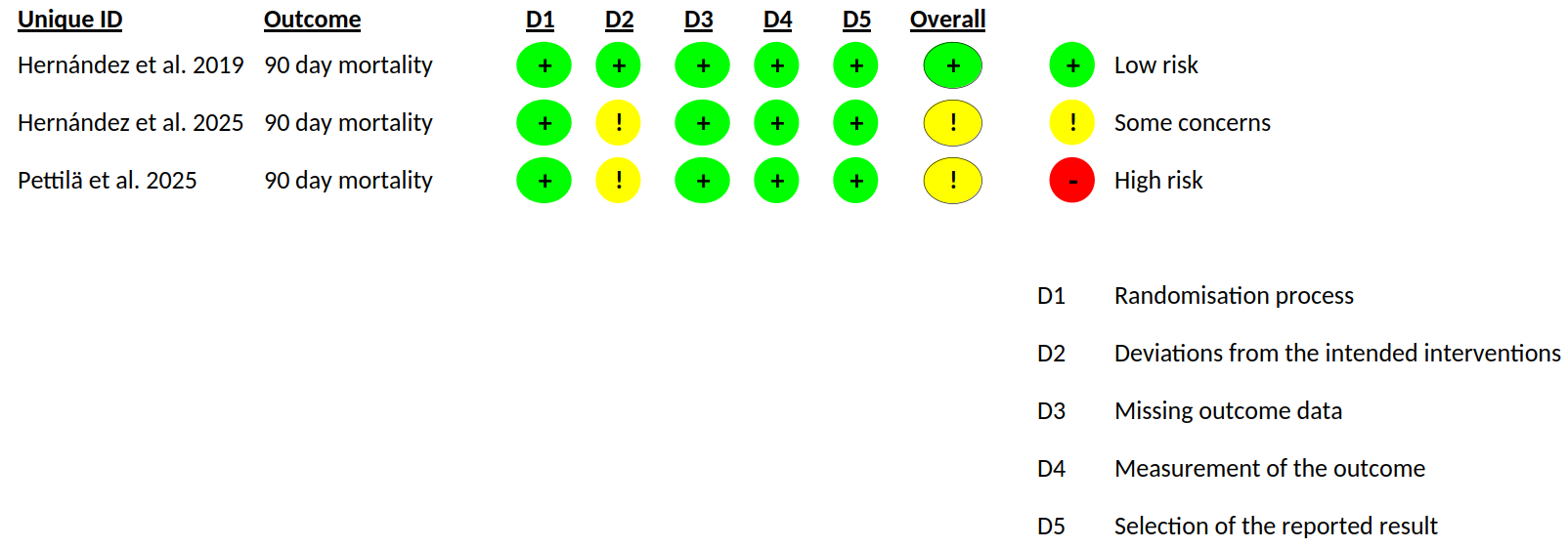
**

**Figure S36.** Risk of Bias assessment for intensive care unit length of stay.

**
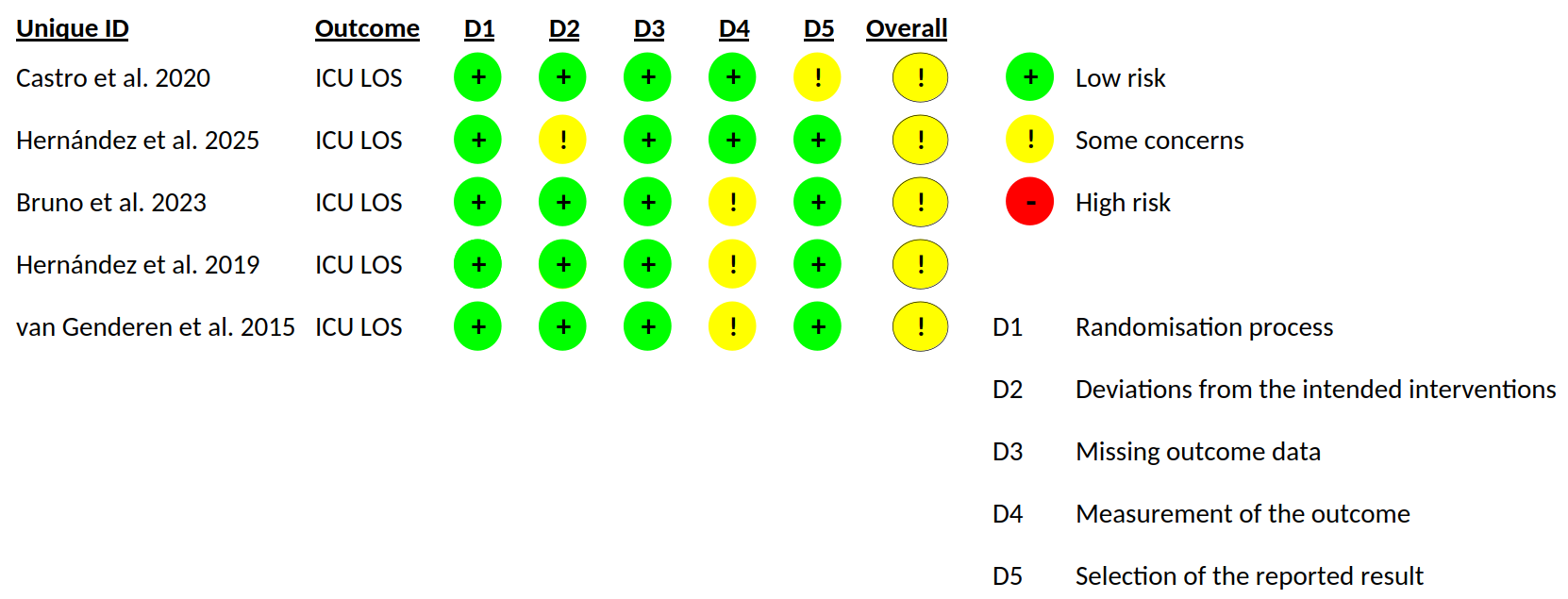
**

**Figure S37.** Risk of Bias assessment for hospital length of stay.

**
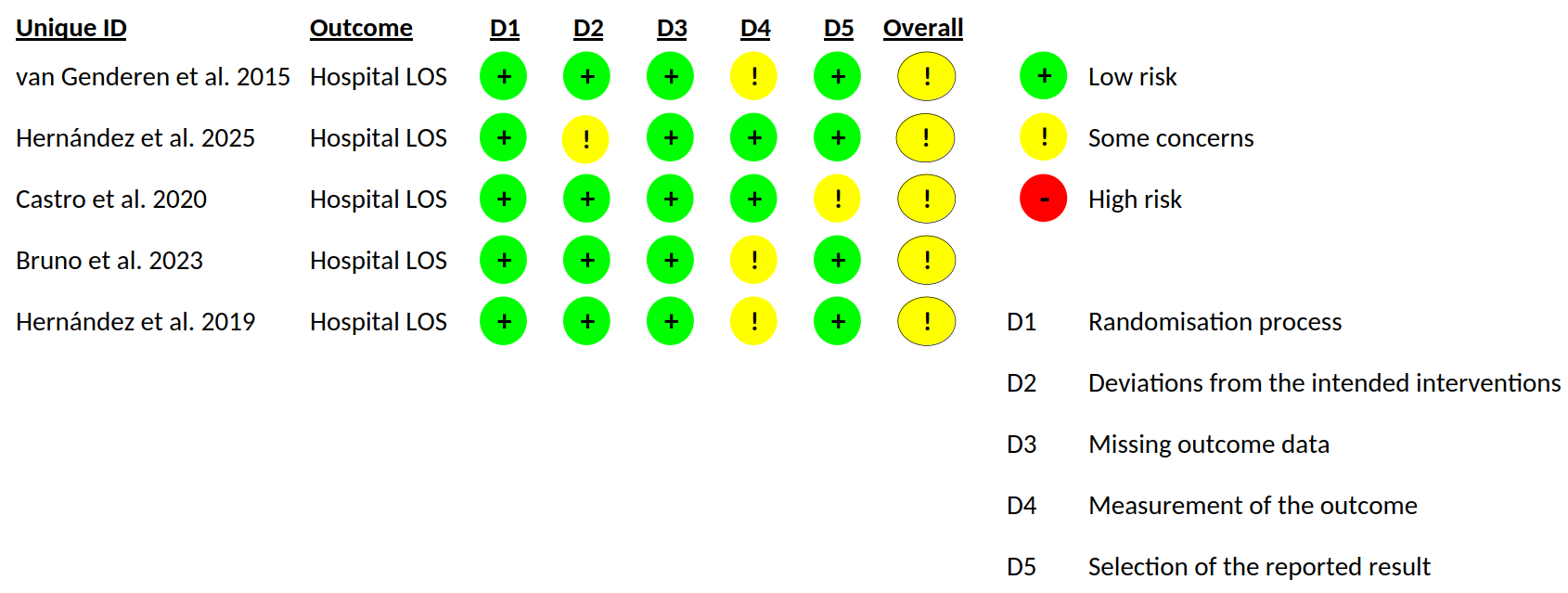
**

**Figure S38.** Risk of Bias assessment for total fluid administered until the end of the 6–8 hour treatment period.

**
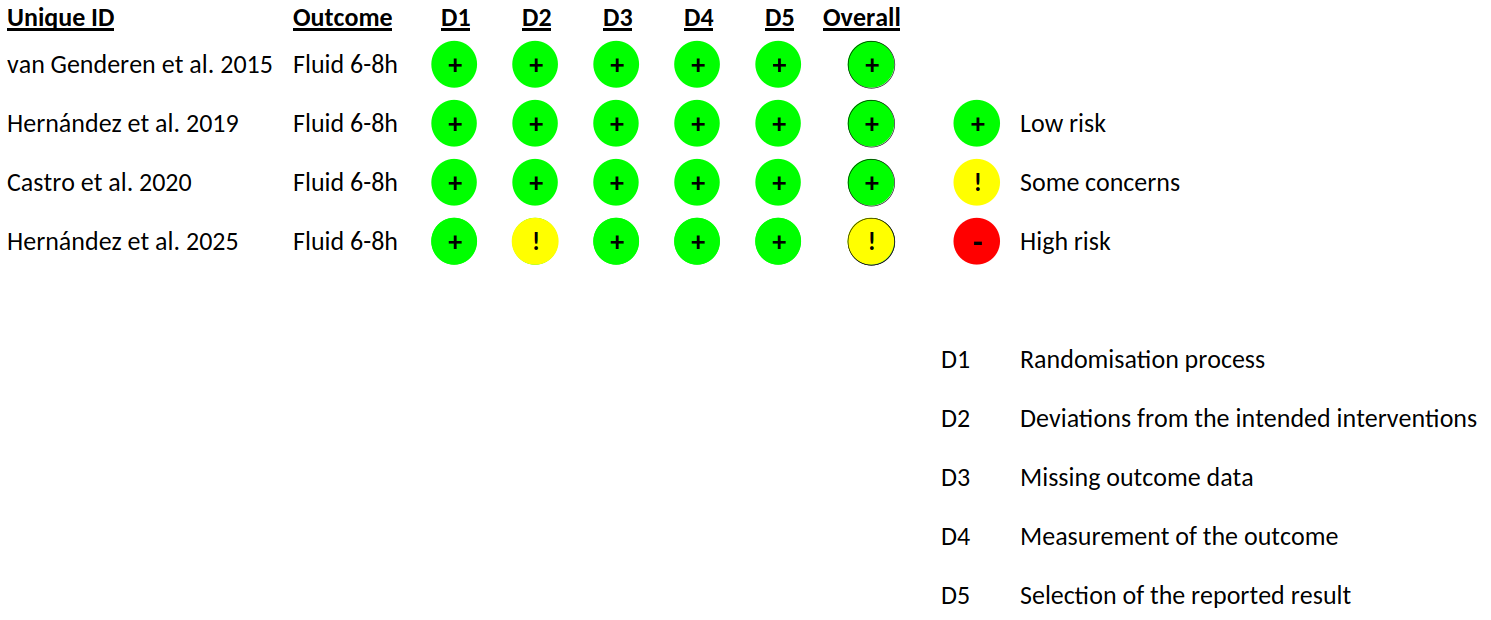
**

**Figure S39.** Risk of Bias assessment for fluid balance until the end of the 24-hour treatment period.

**
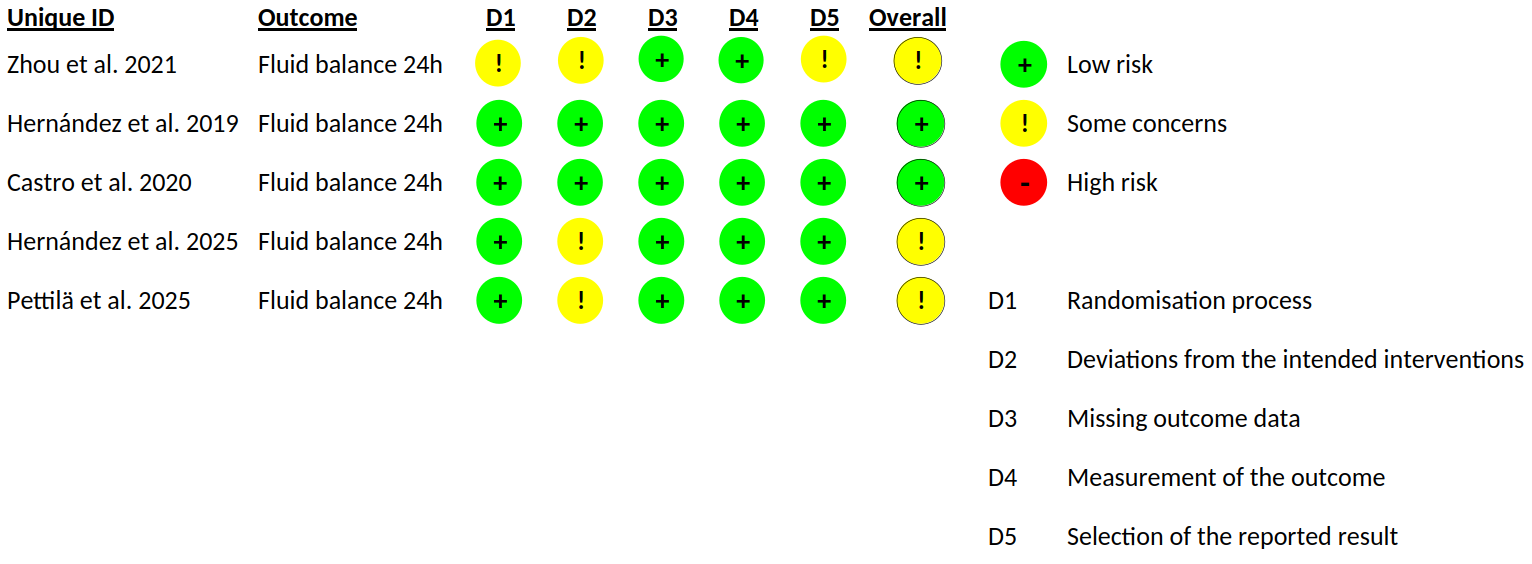
**

**Figure S40.** Risk of Bias assessment for vasopressor-free days.

**
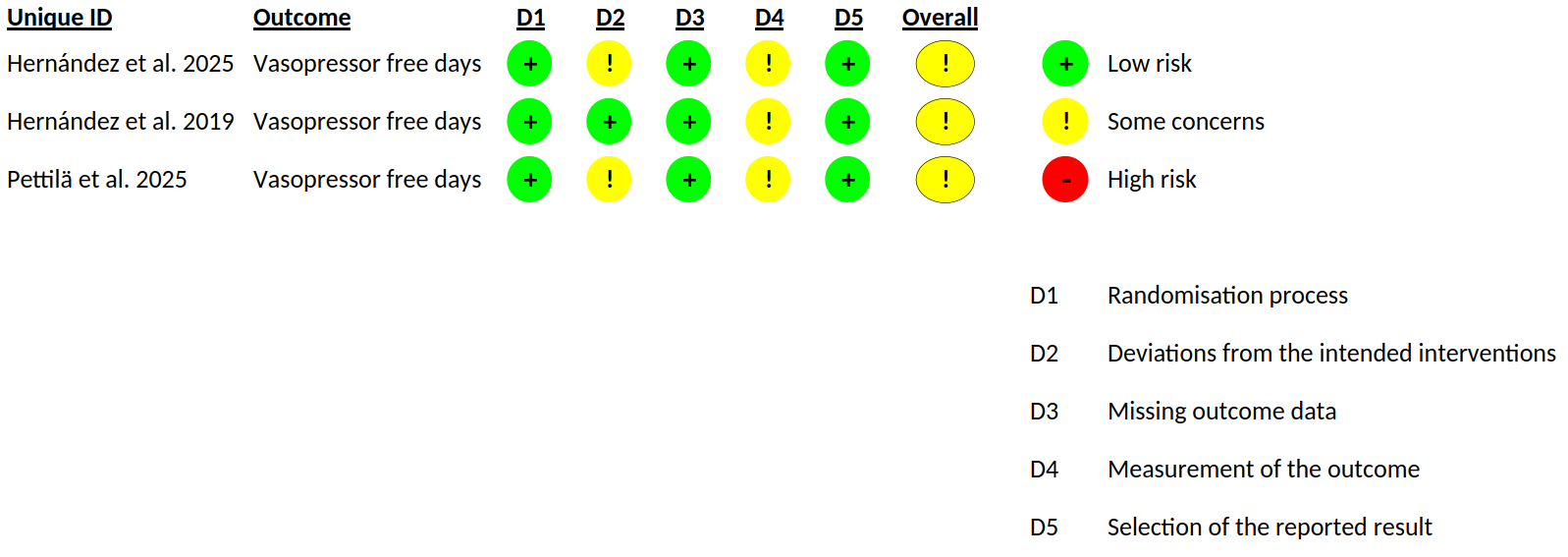
**

**Figure S41.** Risk of Bias assessment for mechanical ventilation free days.

**
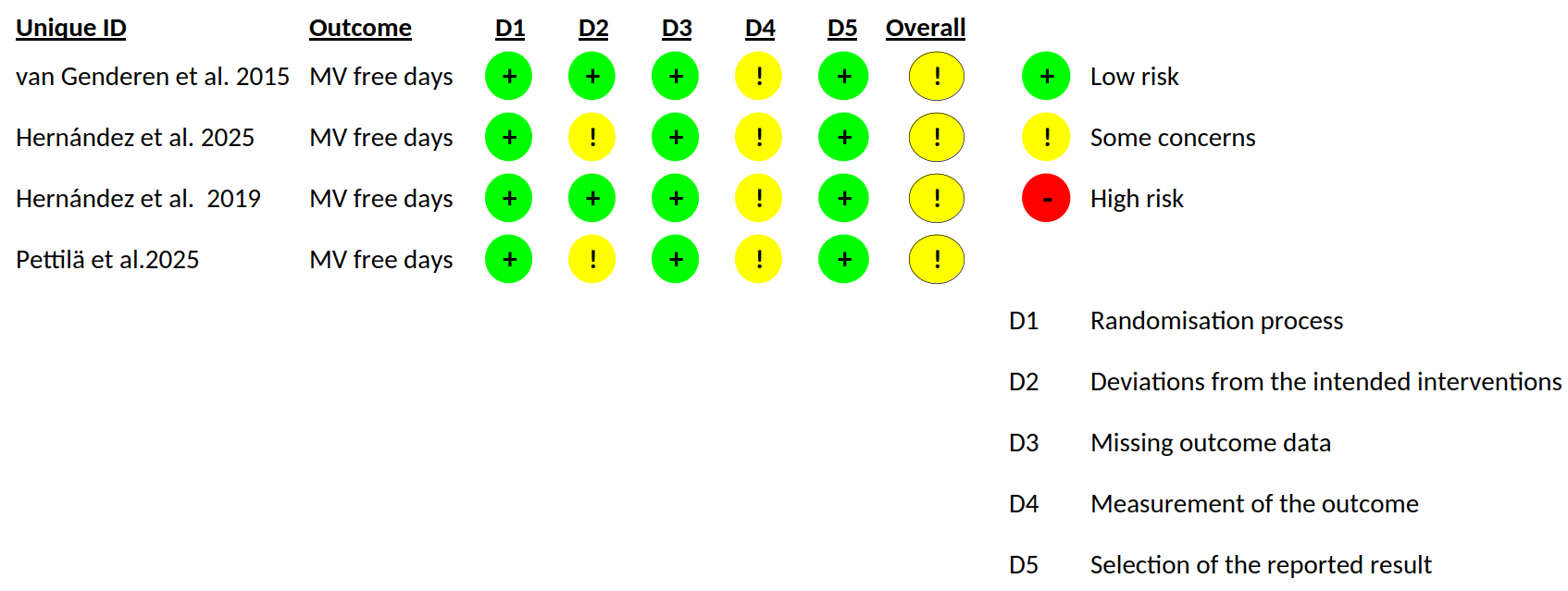
**

**Figure S42.** Risk of Bias assessment for need for renal replacement therapy.

**
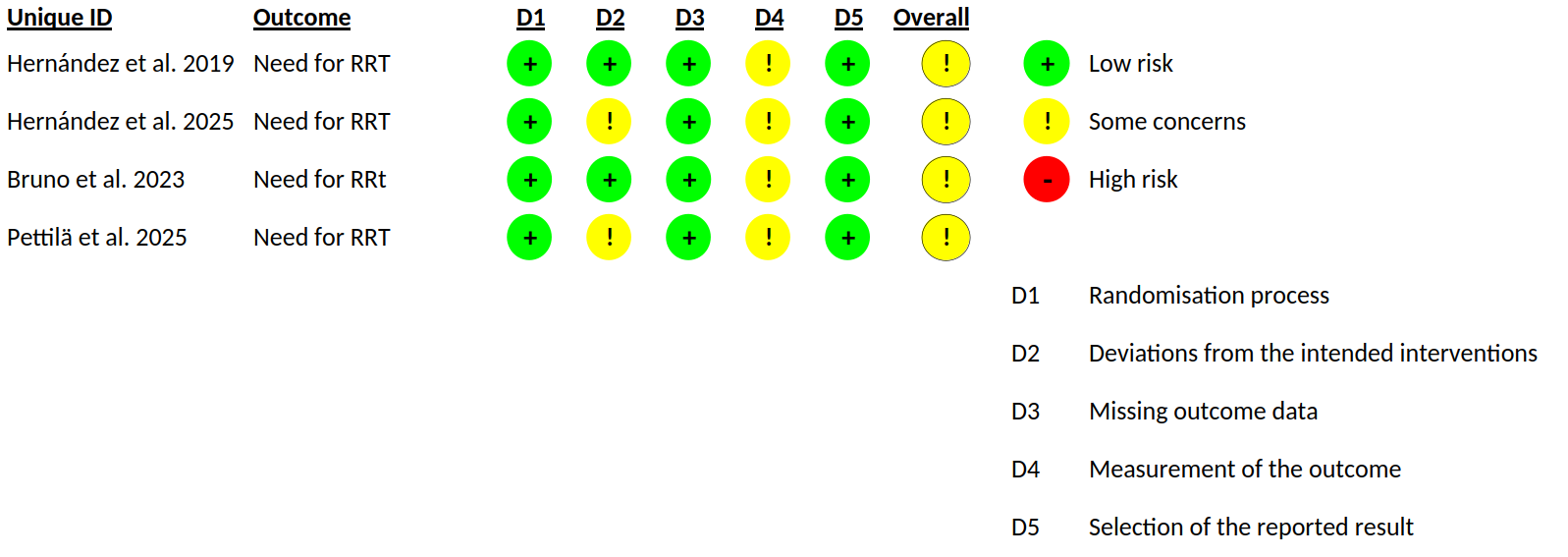
**

**Figure S43.** Risk of Bias assessment for 72h SOFA change.

**
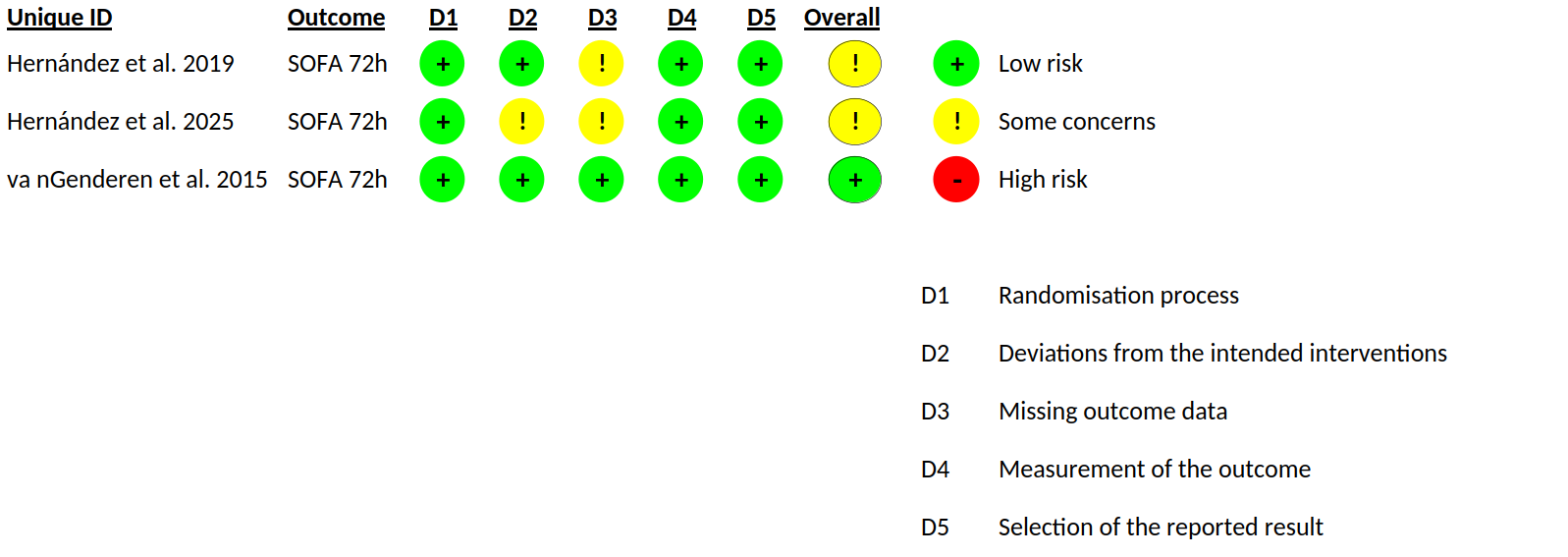
**

**Figure S44.** GRADE assessment.


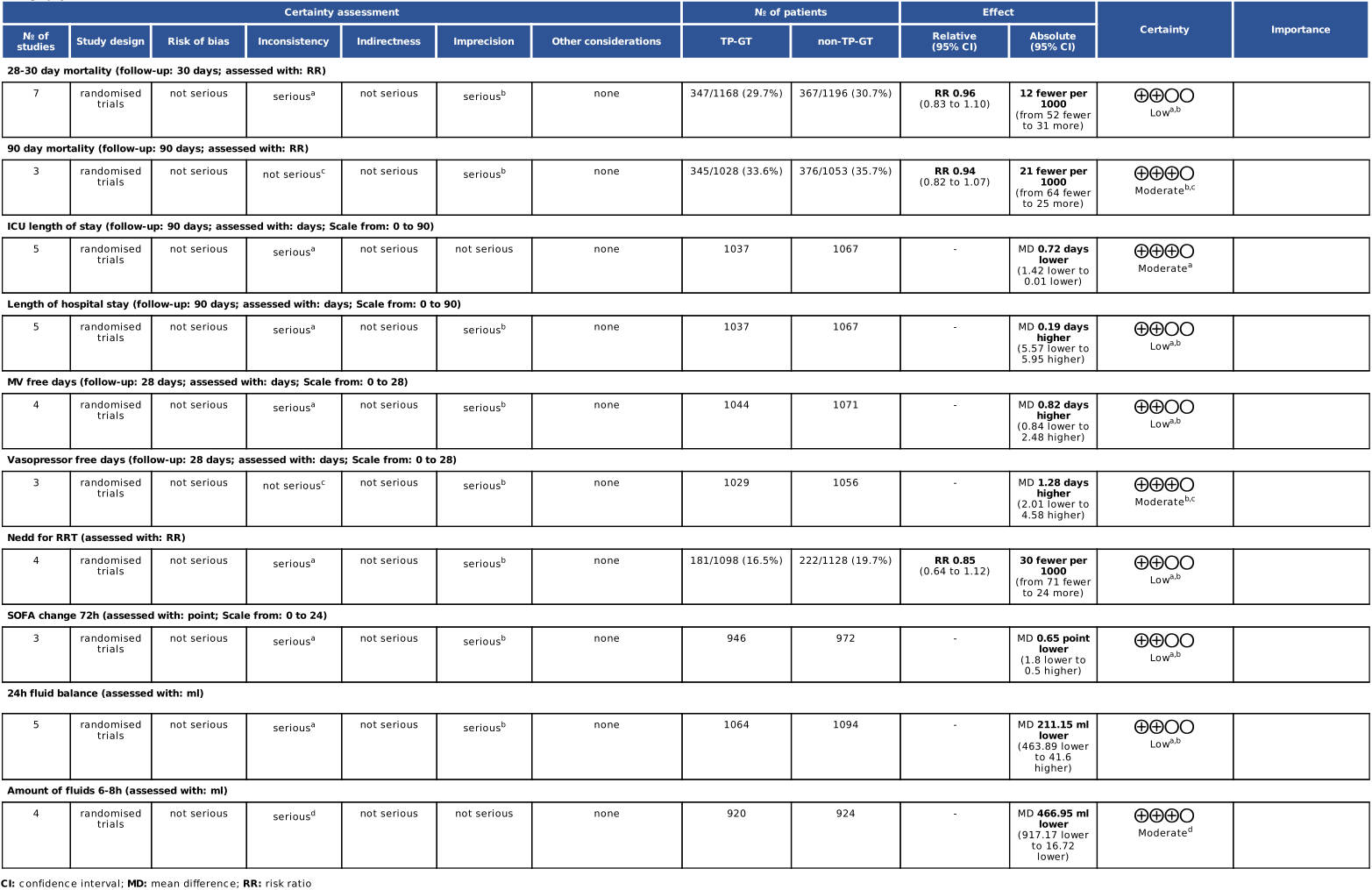


Explanations:

a. Studies are on both sides of the zero effect line.

b. The 95% confidence interval is wide and crosses the line of no effect. Point estimates for all studies consistently favor the intervention group with overlapping confidence intervals.

d. The I^2^ test showed moderate heterogeneity.

**Figure S45.** PRISMA 2020 checklist

| **Section and Topic** | **Item #** | **Checklist item** | **Location where item is reported** |
| --- | --- | --- | --- |
| **TITLE** | | |  |
| Title | 1 | Identify the report as a systematic review. | Article Page 1 |
| **ABSTRACT** | | |  |
| Abstract | 2 | See the PRISMA 2020 for Abstracts checklist. | Article Page 3 |
| **INTRODUCTION** | | |  |
| Rationale | 3 | Describe the rationale for the review in the context of existing knowledge. | Article Page 4 |
| Objectives | 4 | Provide an explicit statement of the objective(s) or question(s) the review addresses. | Article Page 4 |
| **METHODS** | | |  |
| Eligibility criteria | 5 | Specify the inclusion and exclusion criteria for the review and how studies were grouped for the syntheses. | Article Page 5 |
| Information sources | 6 | Specify all databases, registers, websites, organisations, reference lists and other sources searched or consulted to identify studies. Specify the date when each source was last searched or consulted. | Article Page 5 |
| Search strategy | 7 | Present the full search strategies for all databases, registers and websites, including any filters and limits used. | Suppl. Page 4 |
| Selection process | 8 | Specify the methods used to decide whether a study met the inclusion criteria of the review, including how many reviewers screened each record and each report retrieved, whether they worked independently, and if applicable, details of automation tools used in the process. | Article Page 5 |
| Data collection process | 9 | Specify the methods used to collect data from reports, including how many reviewers collected data from each report, whether they worked independently, any processes for obtaining or confirming data from study investigators, and if applicable, details of automation tools used in the process. | Article Page 5 |
| Data items | 10a | List and define all outcomes for which data were sought. Specify whether all results that were compatible with each outcome domain in each study were sought (e.g. for all measures, time points, analyses), and if not, the methods used to decide which results to collect. | Article Page 5 |
|  | 10b | List and define all other variables for which data were sought (e.g. participant and intervention characteristics, funding sources). Describe any assumptions made about any missing or unclear information. | Article Page 5, 6 |
| Study risk of bias assessment | 11 | Specify the methods used to assess risk of bias in the included studies, including details of the tool(s) used, how many reviewers assessed each study and whether they worked independently, and if applicable, details of automation tools used in the process. | Article Page 6 |
| Effect measures | 12 | Specify for each outcome the effect measure(s) (e.g. risk ratio, mean difference) used in the synthesis or presentation of results. | Article Page 6 |
| Synthesis methods | 13a | Describe the processes used to decide which studies were eligible for each synthesis (e.g. tabulating the study intervention characteristics and comparing against the planned groups for each synthesis (item #5)). | Article Page 6 |
|  | 13b | Describe any methods required to prepare the data for presentation or synthesis, such as handling of missing summary statistics, or data conversions. | Article Page 6, 11 |
|  | 13c | Describe any methods used to tabulate or visually display results of individual studies and syntheses. | Article Page 6 |
|  | 13d | Describe any methods used to synthesize results and provide a rationale for the choice(s). If meta-analysis was performed, describe the model(s), method(s) to identify the presence and extent of statistical heterogeneity, and software package(s) used. | Article Page 6 |
|  | 13e | Describe any methods used to explore possible causes of heterogeneity among study results (e.g. subgroup analysis, meta-regression). | Article Page 6 |
|  | 13f | Describe any sensitivity analyses conducted to assess robustness of the synthesized results. | Article Page 14,15,20 |
| Reporting bias assessment | 14 | Describe any methods used to assess risk of bias due to missing results in a synthesis (arising from reporting biases). | Article Page 6 |
| Certainty assessment | 15 | Describe any methods used to assess certainty (or confidence) in the body of evidence for an outcome. | Article Page 6 |
| **RESULTS** | | |  |
| Study selection | 16a | Describe the results of the search and selection process, from the number of records identified in the search to the number of studies included in the review, ideally using a flow diagram. | Article Page 6, 7 |
|  | 16b | Cite studies that might appear to meet the inclusion criteria, but which were excluded, and explain why they were excluded. | NA |
| Study characteristics | 17 | Cite each included study and present its characteristics. | Article Page 6 |
| Risk of bias in studies | 18 | Present assessments of risk of bias for each included study. | Suppl. Page 12-21 |
| Results of individual studies | 19 | For all outcomes, present, for each study: (a) summary statistics for each group (where appropriate) and (b) an effect estimate and its precision (e.g. confidence/credible interval), ideally using structured tables or plots. | Article Page 10, 11  Suppl. Page 5-11 |
| Results of syntheses | 20a | For each synthesis, briefly summarise the characteristics and risk of bias among contributing studies. | Article Page 7, 12 |
|  | 20b | Present results of all statistical syntheses conducted. If meta-analysis was done, present for each the summary estimate and its precision (e.g. confidence/credible interval) and measures of statistical heterogeneity. If comparing groups, describe the direction of the effect. | Article Page 10-12  Suppl. Page 5-11 |
|  | 20c | Present results of all investigations of possible causes of heterogeneity among study results. | Article Page 14,15,20 |
|  | 20d | Present results of all sensitivity analyses conducted to assess the robustness of the synthesized results. | Article Page 14,15,20 |
| Reporting biases | 21 | Present assessments of risk of bias due to missing results (arising from reporting biases) for each synthesis assessed. | Article Page 12 |
| Certainty of evidence | 22 | Present assessments of certainty (or confidence) in the body of evidence for each outcome assessed. | Suppl. Page 22 |
| **DISCUSSION** | | |  |
| Discussion | 23a | Provide a general interpretation of the results in the context of other evidence. | Article Page 12,13 |
|  | 23b | Discuss any limitations of the evidence included in the review. | Article Page 13 |
|  | 23c | Discuss any limitations of the review processes used. | Article Page 13 |
|  | 23d | Discuss implications of the results for practice, policy, and future research. | Article Page 14 |
| **OTHER INFORMATION** | | |  |
| Registration and protocol | 24a | Provide registration information for the review, including register name and registration number, or state that the review was not registered. | Article Page 4 |
|  | 24b | Indicate where the review protocol can be accessed, or state that a protocol was not prepared. | Article Page 4 |
|  | 24c | Describe and explain any amendments to information provided at registration or in the protocol. | Article Page 4 |
| Support | 25 | Describe sources of financial or non-financial support for the review, and the role of the funders or sponsors in the review. | Article Page 2 |
| Competing interests | 26 | Declare any competing interests of review authors. | Article Page 2 |
| Availability of data, code and other materials | 27 | Report which of the following are publicly available and where they can be found: template data collection forms; data extracted from included studies; data used for all analyses; analytic code; any other materials used in the review. | Article Page 2 |

*From:*  Page MJ, McKenzie JE, Bossuyt PM, Boutron I, Hoffmann TC, Mulrow CD, et al. The PRISMA 2020 statement: an updated guideline for reporting systematic reviews. BMJ 2021;372:n71. doi: 10.1136/bmj.n71. This work is licensed under CC BY 4.0. To view a copy of this license, visit <https://creativecommons.org/licenses/by/4.0/>

**Figure S46.** Clinical implications of tissue-perfusion-guided therapy (TP-GT) visualized using the Academia Europaea ring diagram model.

**
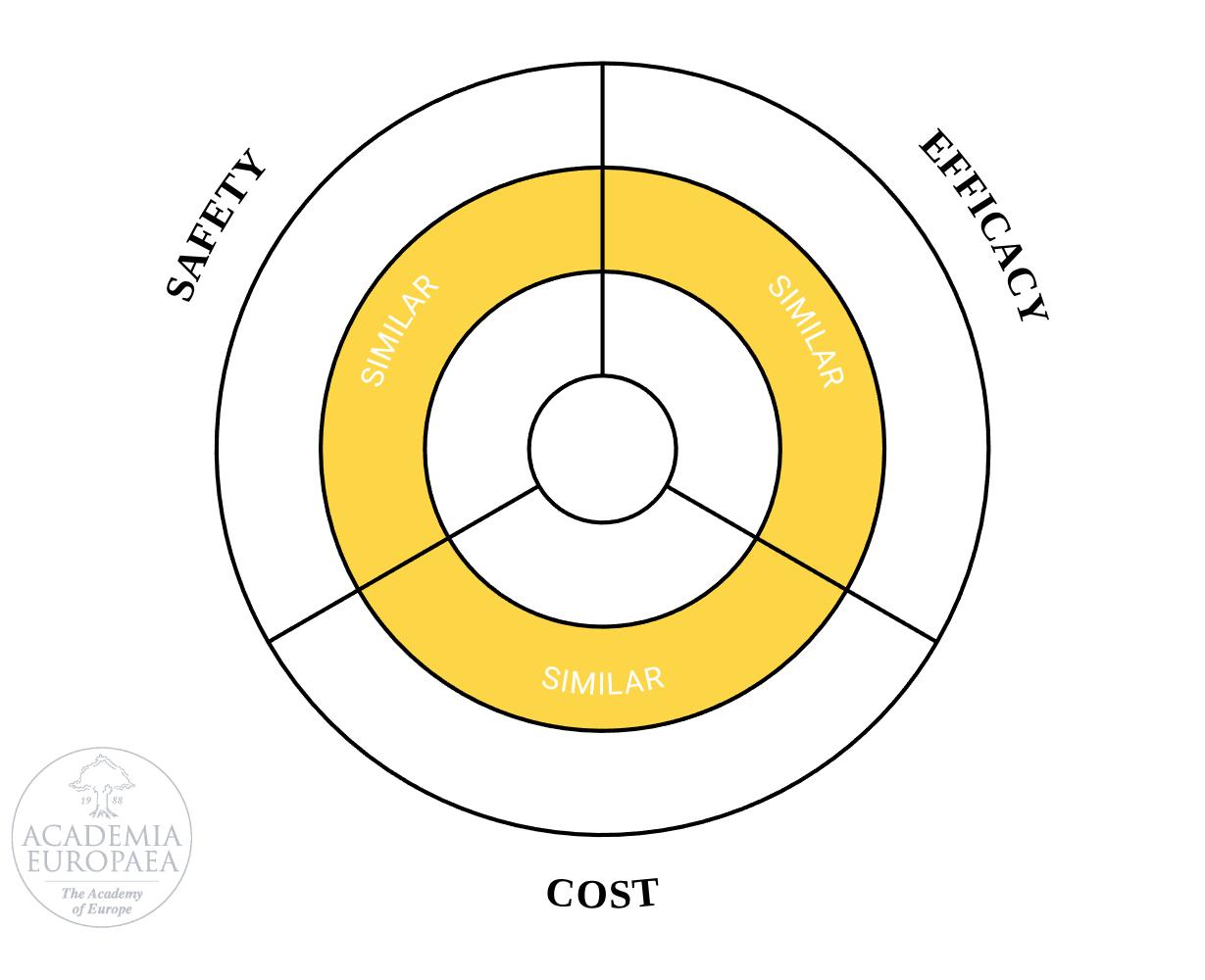
**
